# Continental Patterns of Phenotypic Variation Along Replicated Urban Gradients: A Mega‐Analysis

**DOI:** 10.1111/ele.70180

**Published:** 2025-07-24

**Authors:** M. J. Thompson, J. G. A. Martin, C. Biard, J. Bleu, C. J. Branston, P. Capilla‐Lasheras, N. J. Dingemanse, D. M. Dominoni, M. Eens, T. Eeva, K. L. Evans, C. Isaksson, A. Liker, S. Massemin, E. Matthysen, A. Mouchet, S. Perret, J. C. Senar, G. Seress, M. Szulkin, E. Vincze, H. Watson, D. Réale, A. Charmantier

**Affiliations:** ^1^ Département des Sciences Biologiques Université du Québec à Montréal Montréal QC Canada; ^2^ Centre d'Ecologie Fonctionnelle et Evolutive Univ Montpellier, CNRS, EPHE, IRD Montpellier France; ^3^ Department of Biology University of Ottawa Ottawa Ontario Canada; ^4^ Sorbonne Université, Université Paris Cité, Univ Paris Est Créteil, CNRS, IRD, INRAE, Institut d'écologie et des sciences de l'environnement de Paris, IEES France; ^5^ Université de Strasbourg, CNRS, Institut Pluridisciplinaire Hubert Curien, IPHC UMR 7178 France; ^6^ School of Biodiversity, One Health and Veterinary Medicine University of Glasgow Glasgow UK; ^7^ School of Health and Life Sciences University of the West of Scotland Lanarkshire UK; ^8^ Swiss Ornithological Institute Sempach Switzerland; ^9^ Doñana Biological Station (CSIC) Seville Spain; ^10^ Behavioural Ecology, Faculty of Biology LMU Munich Planegg‐Martinsried Germany; ^11^ Department of Biology University of Antwerp Wilrijk Belgium; ^12^ Department of Biology University of Turku Turku Finland; ^13^ Ecology and Evolutionary Biology, School of Biosciences University of Sheffield Sheffield UK; ^14^ Department of Biology Lund University Lund Sweden; ^15^ HUN‐REN‐PE Evolutionary Ecology Research Group University of Pannonia Hungary; ^16^ Behavioral Ecology Research Group, Center for Natural Sciences University of Pannonia Hungary; ^17^ Departament of Evolutionary and Behavioural Ecology Museu de Ciències Naturals de Barcelona Spain; ^18^ Institute of Evolutionary Biology, Faculty of Biology, Biological and Chemical Research Centre University of Warsaw Warsaw Poland

**Keywords:** blue tit, city, environmental heterogeneity, great tit, individual diversity, intraspecific variation, lay date, subpopulation, tarsus length, urbanisation

## Abstract

Individual variation among and within natural populations can have eco‐evolutionary implications by, for example, affecting species interactions or evolutionary potential. Urban systems present a unique opportunity to evaluate how environmental change shapes variation since urban phenotypic differentiation is widely documented on contemporary timescales. We introduce and test three hypotheses to determine how urbanisation affects phenotypic variation at different population levels. Combining 21 long‐term datasets in a mega‐analysis approach, we synthesise how urbanisation impacts variation in tarsus length and lay date among and within subpopulations of great and blue tits (*Parus major, Cyanistes caeruleus* ) at a continental scale. Our synthesis reveals that urbanisation is associated with increased phenotypic variation within subpopulations by 11% on average, and by as much as 25% across the species and traits examined. We also find some evidence (for tarsus length in great tits) that urbanisation increases differentiation between subpopulations. We did not, however, find that urbanisation increases differences between subpopulations in their within‐subpopulation variation. Our synthesis provides novel insights into how urban contexts impact individual diversity at different spatial scales and we highlight future directions that could establish the genetic and environmental effects that underlie these continental patterns of urban phenotypic variation.

## Graphical abstract

Using a mega‐analysis approach with long‐term data from 21 urban and nonurban great and blue tit populations, we introduce and test hypotheses to determine how urbanisation affects tarsus length and lay date variation at different population levels. We find that urbanisation is associated with increases in phenotypic variation within subpopulations by 11% on average, and some evidence that urbanisation can increase differentiation between subpopulations. Our synthesis provides novel insights into how environmental change modifies diversity at different scales in wild populations.

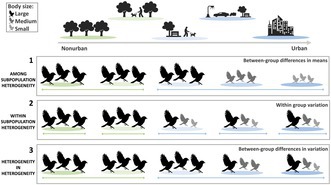

## 1 Introduction

Environmental variation can drive differences between individuals as they adjust or adapt to local conditions and, in turn, these differences may affect how individuals interact with their environment. Thus, phenotypic variation can have important ecological and evolutionary consequences by, for instance, driving individual differences in resource use, predator defence or parasite resistance (Bolnick et al. 2011; Des Roches et al. 2018; Violle et al. 2012; Wolf and Weissing 2012). Investigating how ecological conditions affect phenotypic variation is, thus, an important first step to link sources of phenotypic variation (determined by (epi)genetic variation and plasticity; Falconer 1996; Lynch and Walsh 1998) to evolutionary outputs (e.g., result of selection acting on phenotypic variation; Bürger and Lynch 1995; Ghalambor et al. 2007).

Environmental conditions are dramatically altered in anthropogenic environments (e.g., due to novel resources, modified interactions and urban heat island effects; Szulkin et al. 2020), providing a unique scenario to test how ecological conditions affect phenotypic variation on contemporary timescales. For example, environmental heterogeneity, novel stressors and habitat fragmentation in cities can modify processes of selection, development, (epi)genetic mutation or dispersal with cascading effects on the phenotypic variation contained within urban populations (reviewed in Thompson et al. 2022). Although divergences in mean phenotypes between urban and nonurban populations have been documented across diverse taxa and traits (Diamond and Martin 2021; Lambert et al. 2021; Szulkin et al. 2020), urban and nonurban population differences in phenotypic variation have only recently been studied. In populations of shrews, for example, urban individuals of *Crocidura russula* were found to be more diverse in their aggressiveness and boldness compared to rural individuals (von Merten et al. 2022). In birds, two recent meta‐analyses found that urbanisation increases phenotypic variation in populations for morphological (tit species across Europe; Thompson et al. 2022) and life‐history traits (birds worldwide; Capilla‐Lasheras et al. 2022). Although there is emerging evidence that urbanisation increases phenotypic variation, meta‐analyses are limited to comparing effect sizes across diverse studies. Here we use a more standardised approach that synthesises long‐term datasets (rather than published estimates) providing invaluable replication across space and time, to address new hypotheses and importantly establish how environmental change can modify diversity at different scales in wild populations.

**FIGURE 1:**
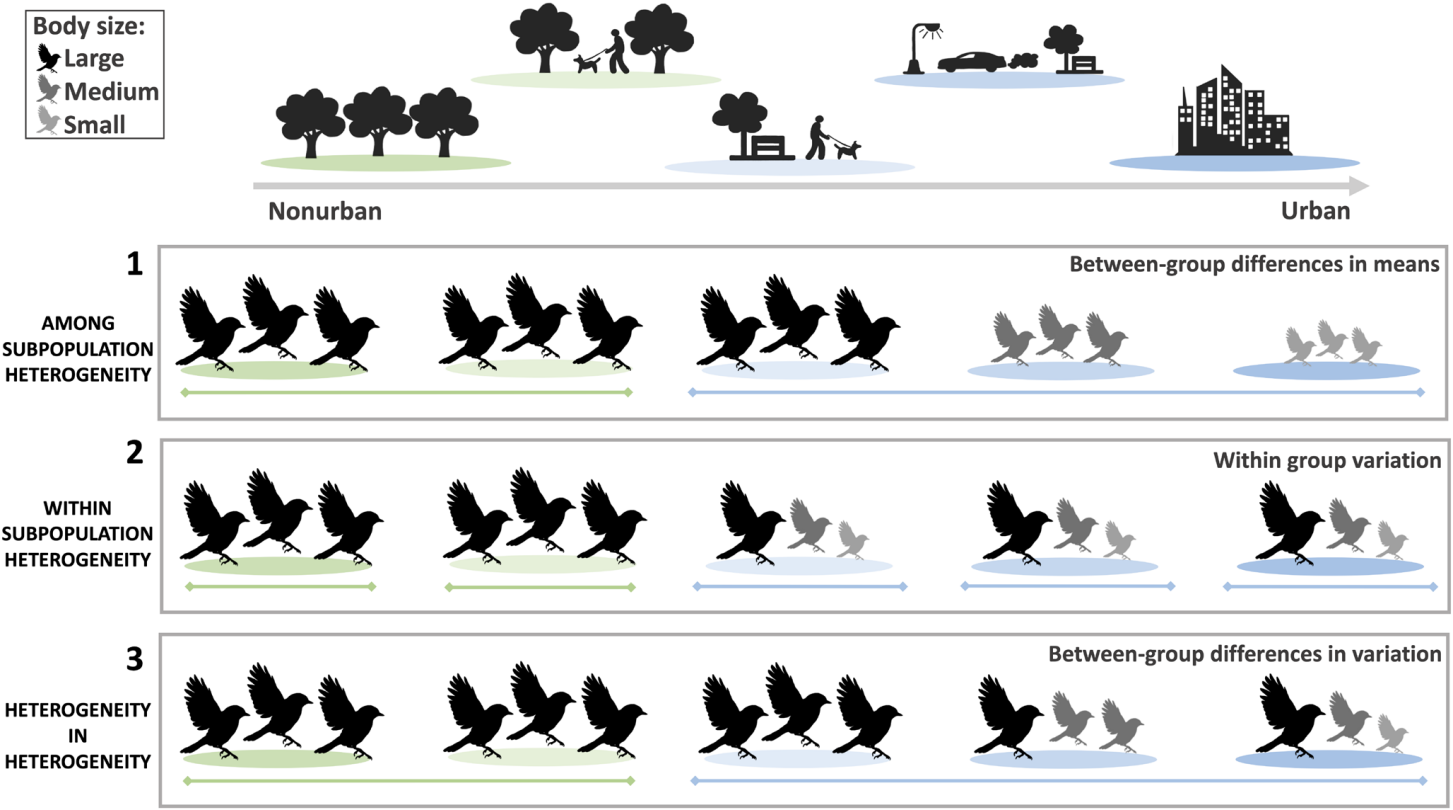
Visual representation of three hypotheses concerning patterns of phenotypic variation among birds in five subpopulations (or clusters as defined in methods) along a theoretical urban gradient where urbanisation increases from left to right. Subpopulations contain groups of nonurban (green) and urban (blue) individuals that differ in body size where individuals are smaller on average in urban subpopulations. Higher phenotypic variation in cities could be driven by (1) among‐subpopulation heterogeneity: Higher differentiation between urban subpopulations since there is higher variation among urban groups in their mean traits than nonurban ones (i.e., some urban subpopulations contain large individuals and others contain medium or small individuals, while nonurban subpopulations all contain large individuals), (2) within‐subpopulation heterogeneity: Higher trait differences within urban subpopulations (i.e., small, medium and large individuals) compared to nonurban subpopulations (i.e., large individuals), (3) heterogeneity in heterogeneity: Differences between urban subpopulations drive higher variation among urban groups in the trait variation they contain (i.e., urban subpopulations contain different compositions of individual sizes).

Ecological processes shape phenotypic variation at different spatial scales in wild populations, for example, by modifying variation both among and within the subpopulations that comprise them (a subpopulation is defined here as a clustered group of individuals occupying the same local environment). We identified three possible hypotheses (H) that could explain recently reported increases in phenotypic variation in urban populations, which we term here the ‘among‐subpopulation heterogeneity’, ‘within‐subpopulation heterogeneity’ and ‘heterogeneity in heterogeneity’ hypotheses (Figure 1). First, under the among‐subpopulation heterogeneity hypothesis (H1, Figure 1,1), we expect increased phenotypic differences between urban subpopulations in cities compared to the phenotypic differences between nonurban subpopulations outside of cities. Cities have been described as more environmentally heterogeneous at larger landscape scales than surrounding areas (Cadenasso et al. 2007; Niemelä et al. 2011; Pickett et al. 2017), which could lead to higher urban variation if subpopulations within a city occupy a wider range of habitats (e.g., city centre vs. urban green space) and are exposed to more diverse conditions than surrounding subpopulations in nonurban habitats (Alberti et al. 2020; Gorton et al. 2018; Rivkin et al. 2019). However, cities have also been referred to as more environmentally homogenous than surrounding areas (Groffman et al. 2014; McKinney 2006; Santangelo et al. 2022) and so changes in urban phenotypic variation will ultimately depend on context (e.g., agents of selection for a given species, trait or scale). Further, human modification of landscapes (e.g., buildings, roads) can reduce dispersal throughout the urban matrix and increase isolation between subpopulations of a species, leading to increasing differences between subpopulations in cities over time (Miles et al. 2019). Indeed, in support of the among‐subpopulation heterogeneity hypothesis there is evidence that urban subpopulations differentiate between each other to a greater extent than subpopulations outside cities, at both the phenotypic (Alarcón‐Ríos et al. 2024; Gorton et al. 2018; Littleford‐Colquhoun et al. 2017) and genetic levels (Miles et al. 2019; Munshi‐South et al. 2016; Schmidt et al. 2020).

**FIGURE 2:**
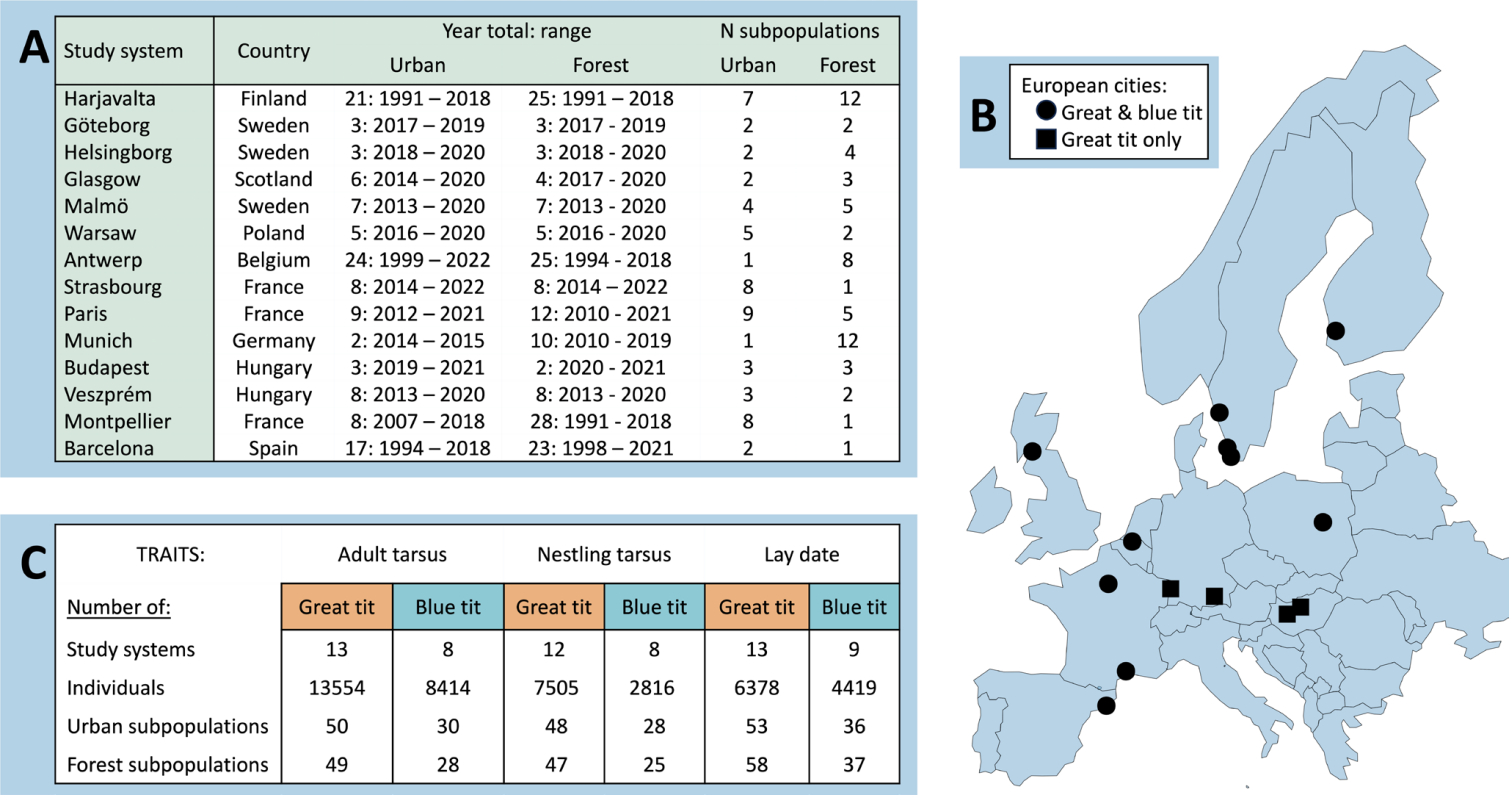
Summary of European urban gradient datasets showing (A) list of study systems (*N* = 14 study systems; listed by decreasing latitude, range = 61°31′ to 41°23′ N) and their year range and number of subpopulations (=clusters adefined by clustering algorithm) in urban and forest habitats, (B) map of Europe showing the location of each study system in A and whether the dataset included great and blue tits (circles) or great tits only (squares) and (C) the three traits examined and the number of study systems, individuals and subpopulations (=urban vs. forest clusters as defined in methods) of the full combined dataset. See also Tables S1 and S2 for further information.

Second, the within‐subpopulation heterogeneity hypothesis (H2, Figure 1,2) predicts higher phenotypic variation within urban subpopulations than nonurban subpopulations, and that each urban subpopulation contains similar amounts of phenotypic variation. Novel resources and stressors can vary at fine spatial and temporal scales even within an urban habitat (Charmantier et al. 2017; Corsini et al. 2019; Jensen et al. 2022; Monniez et al. 2022; Stofberg et al. 2019), which could contribute to specialisations or differences between urban individuals occupying the same local area (Ghalambor et al. 2007). Within a species of terrestrial salamanders ( *Salamandra Salamandra bernardezi* ), some urban subpopulations contained more individual diversity in head shape than forest subpopulations, presumably as urban factors could impair developmental canalisation (Alarcón‐Ríos et al. 2024) and increase morphological deformities in cities (Velo‐Antón et al. 2021).

**FIGURE 3:**
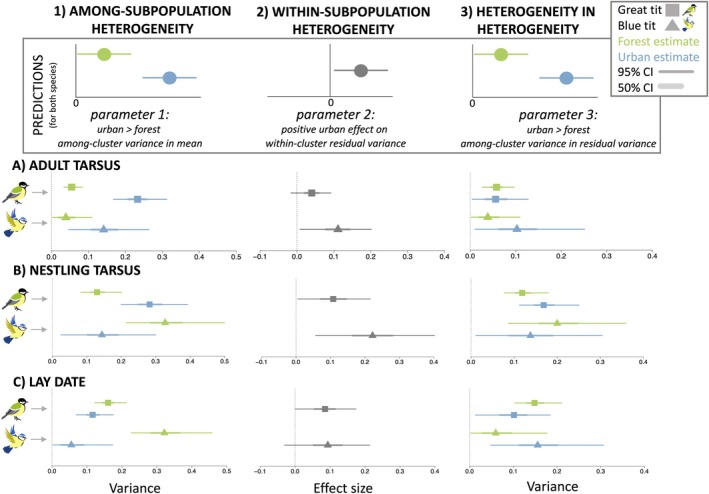
Results of each hypothesis and associated predictions (columns) for (A) adult tarsus length, (B) nestling tarsus length and (C) lay date traits (rows). (1) Among‐subpopulation heterogeneity hypothesis (parameter 1) shows the variance estimated among forest and urban clusters on the mean of the traits, (2) Within‐subpopulation heterogeneity hypothesis (parameter 2) shows the urban effect on the residual variance of the traits (forest versus urban; positive values indicate more residual variation in urban habitats) and (3) Heterogeneity in heterogeneity hypothesis (parameter 3) shows the variance estimated among forest and urban clusters on the residual variance of the traits. Model estimates related to each parameter are from Table 1 and their 95% (thin line) and 50% (thicker line) highest posterior density intervals (or CI = credible interval) are shown. Parameters 1 and 3 are fitted as random effects in the model and return two variance estimates (urban = blue, forest = green), whereas parameter 2 is fitted as a fixed effect and returns a slope coefficient (effect of urbanisation = grey; see also Table 1). See also Figure S8 for results of 1 and 3 presented as the difference between urban and forest variance posterior draws. Estimates for great tits are represented by squares and blue tits by triangles.

Third, the heterogeneity in heterogeneity hypothesis (H3, Figure 1,3) predicts greater differences between urban subpopulations in the phenotypic variation they contain compared with nonurban subpopulations. Environmental heterogeneity or fragmentation in urban contexts may contribute to increases in urban phenotypic variation, but it is possible that these factors and processes like selection, plasticity or dispersal vary considerably in space across the urban matrix. Less uniformity in these factors and processes within cities than surrounding areas may mean that some urban subpopulations contain high phenotypic variation and others low phenotypic variation, contributing to higher differences between subpopulations in their within‐subpopulation variation in urban habitats. For example, it has been suggested that higher environmental heterogeneity within cities could lead to spatially variable selection pressures (Rivkin et al. 2019) where relaxed selection could increase phenotypic variation within some urban subpopulations and stronger selection could reduce phenotypic variation within other urban subpopulations, while selection pressures and resulting variation within nonurban subpopulations remain similar. To our knowledge, however, this third hypothesis is yet to be explicitly introduced and tested. These three hypotheses are not mutually exclusive and determining how urbanisation increases phenotypic variation both among and within subpopulations will facilitate inferences towards which population level processes like selection, plasticity or dispersal modify variation in urban contexts.

Here we synthesise how phenotypic variation is spatially distributed along replicated urban gradients by partitioning phenotypic variation at different population levels in two European tit species (great tits, *Parus major* and blue tits, *Cyanistes caeruleus* ). To build on findings from previous meta‐analyses (Capilla‐Lasheras et al. 2022; Thompson et al. 2022) we use a mega‐analysis (Eisenhauer 2021; Koile and Cristia 2021; Sung et al. 2014) based on a large‐scale European collaboration (here 21 populations, 157 subpopulations and 21,968 individuals across 2 species, Figure 2). While a meta‐analysis uses pooled summary statistics from previous studies, a mega‐analysis is based on the pooled standardised raw data from multiple independent studies (Eisenhauer 2021; Koile and Cristia 2021; Sung et al. 2014). Although mega‐analyses are ambitious approaches often requiring coordination and collaboration across different research groups, this comprehensive approach allowed us to examine finer‐scale patterns of phenotypic variation across replicated urbanisation gradients with the additional benefits of (i) accounting for variance in the combined dataset in a single statistical model and (ii) ideally involving all data owners throughout the research process. Great tits and blue tits are commonly studied in both urban and nonurban habitats, and the availability of long‐term datasets obtained using highly standardised methodology on these species provides a unique opportunity to address our three hypotheses and generalise our results in populations across most of the species' distributions. Our study focuses on variation in tarsus length and lay date. It has been recently shown that urban bird populations tend to contain more variation in these traits than nonurban populations (Capilla‐Lasheras et al. 2022; Thompson et al. 2022), but which of our hypotheses lead to these global patterns is unknown.

We predict that higher urban variation will be driven by a combination of higher differentiation between urban subpopulations (Hypothesis 1; Figure 1,1) and higher variation within urban subpopulations (Hypothesis 2; Figure 1,2) for both tarsus length and lay date. Less is known about how variation changes across the urban matrix and so we explore whether differences between urban subpopulations in the variation they contain could also contribute to variation in these traits (Hypothesis 3; Figure 1,3). As great tits and blue tits are closely related species with similar niches, we expect parallel responses to urbanisation and make similar predictions for both species. Besides comparing trait variation between categorically classified urban and nonurban subpopulations, we also quantify urbanisation in a standardised way across all European populations to consider how the degree of urbanisation impacts trait means and variation at small and large spatial scales. Our results provide important insights on the eco‐evolutionary processes that shape phenotypic variation in urban contexts, and we highlight outstanding knowledge gaps that can be addressed moving forward.

## 2 Methods

### 2.1 Datasets and Phenotypic Traits

Urban and nonurban populations of great tits and blue tits have been monitored as a part of long‐term research programmes that involve following the reproduction of individuals in nest boxes. We combined datasets from 14 urban gradients (cities and surrounding area; we term this here ‘study system’) across Europe (Figure 2). We obtained three full standardised datasets from the SPI‐Birds network (Culina et al. 2020) and the remaining datasets were received directly from data owners. These datasets varied greatly in the number of years of data collection, the number of subpopulations and nest boxes along each gradient (Figure 2), and the data collected for each species and trait (Figures S1–S3).

We focused on two phenotypic traits: tarsus length and lay date. Tarsus length is fixed early in life (i.e., end of post‐hatching growth before fledging; Björklund 1997; Gebhardt‐Henrich and Van Noordwijk 1994) and is an indicator of body size or competitive ability in tits (Kempenaers et al. 1992; Oddie 2000). We compared among‐ and within‐subpopulation variation in tarsus length between nestling and adult life stages since similarities or contrasts between patterns could reveal clues about the processes (e.g., selection via post‐fledging survival) shaping tarsus variation in urban contexts. For tarsus length, we used individual‐level data from capture events during the breeding season from both adults and nestlings (adults: individuals that hatched before the current year and nestlings: the current year). Nestlings were measured on average 15 days (range: 13–17) after hatching before they fledged the nest. Individual tarsus length was measured using the Svensson's Alternative Method (Svensson 1992) or converted to this method if measured differently (see Supporting Information Methods 1.1 and Equations S1, S2).

In contrast, a female's laying date is highly plastic to annual environmental conditions like spring temperature and habitat phenology (Bourgault et al. 2010; Visser et al. 2009), even at small spatial scales (Cole et al. 2021; Hinks et al. 2015), and can have major implications for reproductive success (Marrot et al. 2018; Nager and van Noordwijk 1995). For lay date, nest boxes were monitored regularly during the breeding season and the lay date of each female was determined as the date when the first egg of a clutch was laid (range: March 16–June 1; 75–152 Julian days). We only considered nestling tarsus and lay dates from first clutches as not all datasets included monitoring of second broods, and the breeding season length and frequency of double brooding differ across Europe and between urban and nonurban habitats (Bukor et al. 2022; Sinkovics et al. 2023; Verboven et al. 2001; Visser et al. 2003). First clutches were defined as the first clutch laid by a female in a breeding season and were within 30 days of the first lay date of a focal species in a focal year within a focal subpopulation (Culina et al. 2020; Van Balen 1973).

We chose to evaluate phenotypic variation at population levels rather than the individual level since repeated individual measures in the full dataset were low (approx. 1.4 measures per adult) and could limit the ability to estimate within‐individual variation. We hence included only one observation per individual to estimate individual differences between and within subpopulations, while avoiding pseudoreplication in statistical models (see Supporting Information Methods 1.2–1.4 for further justification of which measures were used). Since adult tarsus length should not change over repeated measures, we computed the mean tarsus length for each individual if they had more than one measure to reduce measurement error. To minimise model complexity for nestling tarsus length and ease comparability with results of adult tarsus length, we followed a similar approach for nestling tarsus by selecting one random nestling from each nest box within each year (one nestling per brood). We selected only the first appearance of each female with known identity in the dataset and their corresponding lay date. A summary of the combined datasets for the three traits is shown in Figure 2C.

### 2.2 Environmental Variables

As study areas defined by data owners differed in shape and size across study systems, we aimed to standardise the definition of a subpopulation by defining clusters of nest boxes (*N* = 119 clusters; Tables S1 and S2). We use the term ‘cluster’ here to represent subpopulations along urban gradients in this study (as shown in Figure 1), which were defined by a clustering algorithm to contain at least 5 nest boxes within a 300‐m distance of each other that tended to be contained in the same habitat type (for our rationale see Supporting Information Methods 1.5; Table S3). Although sampling varied across clusters in the dataset (Figures S1–S3) and clusters with lower sample sizes could have inflated variance by chance, our mixed modelling approach (see Statistical approach) allows robustness to this unbalanced sampling.

We examined urbanisation at each cluster as (i) categorical (urban vs. forest) and (ii) continuous by quantifying impervious surface area (ISA) at a small and large spatial scale (100 and 1000 m; see Supporting Information Methods 1.5 and Figure S4). We defined the habitat category of each cluster following data owners' categorisation, which was consistent across study systems; urban clusters were within close proximity to city centres with higher average ISA (0.46 proportion ISA at 100 m) and forest clusters were in forested areas outside cities with lower average ISA (0.01 proportion ISA at 100 m). Examining urbanisation more generally (categorical: combines imperviousness, human presence, light, supplementary food, etc.) and via the reduction of natural habitat (continuous: ISA) allowed us to determine whether these urbanisation effects had similar impacts on phenotypic variation. We also quantified large‐scale land cover heterogeneity for each cluster using the Shannon Diversity Index of land cover classifications (see Supporting Information Methods 1.5 and Figure S5).

### 2.3 Statistical Approach

We used double hierarchical generalised linear models, or DHGLMs, which are an extension of mixed models that quantify how residual variance varies among groups (Cleasby et al. 2015; O'Dea et al. 2022). In ecology and evolution, these models have been used to quantify between‐individual differences in intra‐individual residual variance or ‘individual predictability’ (i.e., how consistent an individual is in a trait; Hertel et al. 2021; Martin et al. 2017). These models require repeated observations per unit of analysis to estimate whether units are predictable and show low variation around their average characteristic, or unpredictable and show high variation. Here, we used clusters (i.e., subpopulations) instead of individuals as the lowest grouping level, taking only one observation per individual or nestling per brood so that each cluster contained observations from multiple individuals. In this context, we could evaluate how ‘predictable’ or variable individuals sampled in different clusters are and if this phenotypic variation could be explained by environmental variables like urbanisation. Thus, within‐cluster variation includes both among‐ and within‐individual sources of variation for adult tarsus and lay date, and among‐ and within‐brood variation for nestling tarsus.

We use DHGLMs to estimate three key parameters allowing us to test each of our main hypotheses. First, to evaluate the among‐subpopulation heterogeneity hypothesis (H1, Figure 1,1), we examined whether urban clusters' means are more variable than forest ones (parameter 1: among‐cluster variance in mean trait; prediction: urban > forest). Second, to evaluate the within‐subpopulation heterogeneity hypothesis (H2, Figure 1,2), we determined whether more urbanised clusters contain more phenotypic variation than less urbanised clusters (parameter 2: slope coefficient of urbanisation effect on within‐cluster residual variance; prediction: positive effect). Third, to evaluate the heterogeneity in heterogeneity hypothesis (H3, Figure 1,3), we explored whether variation within clusters differed by habitat type (parameter 3: among‐cluster variance in within‐cluster residual variance; prediction: urban > forest). Thus, both parameter 1 and 3 return two among‐cluster variance estimates for urban and forest groups separately (fitted as random effects), while parameter 2 returns a single coefficient estimating how urbanisation (categorical or continuous) affects variation within clusters (fitted as fixed effect, see model descriptions below).

We fitted DHGLMs using tarsus length or laying date as our response variable in R (v.4.2.1) with the package brms (Bürkner 2017) using the Stan software (Carpenter et al. 2017; Stan Development Team 2023). We fitted fixed and random effects in both the mean (explains mean effects and among‐level variation in mean trait values) and dispersion (explains residual variance) parts of the model to control for known effects on trait means and estimate our three parameters of interest. We fitted the same model structures (described below) for great and blue tits separately to avoid fitting species interactions across model effects and ease interpretation. Response variables of the combined datasets were standardised (*Z*‐transformed; mean = 0, SD = 1) and fitted using a Gaussian distribution. We also standardised all continuous fixed effects to help with model fit and convergence. Since our response variables were *Z*‐transformed (Hertel et al. 2021; McElreath 2020), we used weakly informative normal priors [*N*(location mean = 0, scale = 1)] for fixed effects, half‐normal priors for random effects [*N*(0,1)] and a Lewandowski‐Kurowicka‐Joe correlation prior (LKJ prior; df = 2) for correlations of random effects. We ran four chains for 10,000 iterations each using a warm‐up of 6000 iterations and a thinning interval of 4. Thus, model estimates and highest posterior density intervals (HPDIs) used posterior distributions consisting of 4000 samples. All models had appropriate convergence with Rhat = 1 and effective sample sizes > 400 (Vehtari et al. 2021), and inspection of model diagnostic plots (traces, residuals, posterior predictive checks) confirmed good model fit.

Following suggestions from the American Statistical Association (Wasserstein and Lazar 2016), we apply a more continuous approach to statistical inference (Dushoff et al. 2019; Held and Ott 2018; Muff et al. 2022). We infer evidence in support of our hypotheses from effects whose 95% highest posterior density interval (HPDI or credible interval) excludes zero. In addition, we infer weak evidence from effects whose 95% HPDIs overlap zero but for which the probability of direction (pd) in support of our hypothesis is > 0.90; where pd is estimated as the proportion of the posterior distribution that is in the direction predicted (Held and Ott 2018). Effects with pd < 0.90 (and 95% HPDI overlapping zero) were interpreted as an absence of evidence.

### 2.4 Adult Tarsus Length

Before analysis, we removed four clear outliers for adult tarsus measures visually outside the range of the other measures (12.70–25.49 mm; both species). Based on previously documented effects on mean tarsus length published on these species (Biard et al. 2017; Caizergues et al. 2021; Corsini et al. 2021; Saulnier et al. 2023; Seress et al. 2020; Thompson et al. 2022) we included the fixed effects of cluster habitat type (urban vs. forest), mean latitude of each cluster (range = 41°23′—61°31′ N) and sex (female or male) in the mean part of the model. We included random intercept effects for each study system (i.e., *N* = 13, excludes Harjavalta dataset because of limited adult tarsus length data) and for each sampling year (range = 1991–2022) to estimate among‐system and among‐year variation. To evaluate whether urban clusters differentiate to a greater extent than forest ones in their means (parameter 1; Figure 1,1), we fit cluster variance (i.e., random intercepts) separately for urban and forest habitats, and thus obtained one variance estimate for urban and one for forest clusters.

In the dispersion part of the model, we fit the fixed effect of habitat type (urban vs. forest) to explain tarsus variation within clusters and test whether urban clusters contain more phenotypic diversity than forest clusters (parameter 2; Figure 1,2). Parameter 2 is a single coefficient estimating the difference in the average within‐cluster residual variation between urban and forest habitats. Males are often more variable in their body sizes than females, especially in mammals (Zajitschek et al. 2020), but this trend could be reversed in birds (Reinhold and Engqvist 2013). As great and blue tits are dimorphic species, we also explored potential sex differences in tarsus variation by fitting sex as a fixed effect. We also included the fixed effects of land cover heterogeneity (Shannon diversity) and mean latitude to determine their contributions to variation within clusters. To account for differences between clusters in their size and number of years of data collection on the variation they contain, we also controlled for cluster area (median = 0.12 km^2^, range = 0.0065–226.6; see Supporting Information Methods 1.5) and number of years each cluster was studied (range = 1–28) as fixed effects. We fit random intercepts for study systems to estimate within‐system variation. We then also fit heterogeneous variance for urban and forest clusters in the dispersion model to examine whether urban clusters differ more in their tarsus variation than forest clusters (parameter 3 returns two variance estimates, one for urban and one for forest clusters; Figure 1,3). Following the format presented in O'Dea et al. (2022), we present mathematical model equations of the mean part of the model, the dispersion part of the model and their covariance for the analysis of adult tarsus in Table S9 and Equations S3–S8. As an additional step, we ran Supporting Information Models by replacing the categorical habitat effect with proportion impervious surface area (ISA) at 100‐ and 1000‐m scales in the mean and dispersion parts of the model to evaluate how the mean and variation of tarsus were affected by standardised continuous urbanisation. We did not fit heterogeneous habitat variance for clusters in these models.

### 2.5 Nestling Tarsus Length

We included data from *N* = 12 study systems (excluding Harjavalta and Barcelona because of limited nestling tarsus length data) and removed nine outliers that were visually outside the range of measures of both species for nestling tarsus length (great tits: range = 10.2–25.9 mm, blue tits: range = 12.11–21 mm). The DHGLM model structure for nestling tarsus included the same fixed and random effects in the mean and dispersion parts of the model as for adult tarsus, but, as nestlings are unreliably sexed this early in life, we did not examine the effects of sex. Instead, we controlled for the effect of nestling age on the mean tarsus length (i.e., as a fixed effect in the mean part of the model, range = 13–17 days old).

### 2.6 Lay Date

We included lay date data from *N* = 13 study systems (excluding Barcelona because of limited lay date data) and the DHGLM model structure for lay date contained the same fixed and random effects in the mean and dispersion parts of the model as for adult tarsus, with one exception. As lay date is a female trait, we did not include sex as a predictor in the model. We instead included the age category of female breeders (yearling = 1 year old vs. older = 2+ years of age) as a fixed effect in the dispersion part of the model. As experience and learning could play a role in a female's perception of environmental cues and lay date decisions (Bonamour et al. 2019), we explored whether yearlings or older birds differed in their lay date variation.

## 3 Results

Results related to our three hypotheses and parameters of interest are reported below. See Supporting Information Results (Section 2 and Figures S6 and S7) for reporting on urban divergence in trait means and other model effects.

### 3.1 Among‐Subpopulation Heterogeneity Hypothesis (H1 and Parameter 1)

For adult tarsus length, the urban among‐cluster variance in mean tarsus length was approximately four times higher than the forest among‐cluster variance in both species; urban groups differentiated between each other to a greater extent than forest groups in their mean tarsus lengths (parameter 1: urban > forest among‐cluster variance in mean trait, Tables 1A and S4). We had evidence for this result in great tits (Figures 3,1A and S8: 95% HPDI of difference in variance not overlapping zero), but only weak evidence in blue tits (Figure 3,1A) since the 95% HPDI of difference in variance overlapped zero and posterior direction (pd) = 0.95 (Figure S8). For nestling tarsus in great tits, the among‐cluster variance was twice as high for urban compared to forest clusters (parameter 1: urban > forest among‐cluster variance in Table 1B; Figure 3,1B). However, the pattern for nestling tarsus length differed in blue tits as the among‐cluster variance was slightly higher for forest than urban clusters (Table 1B; Figure 3,1B), and the difference in variance between habitat types had only weak support (pd = 0.96; Table 1B; Figure S8). For lay date in great tits, there was similar among‐cluster variance in mean lay date and largely overlapping HPDIs between the habitat types (parameter 1 great tits: urban ~ forest among‐cluster variance in means, Table 1C; Figures 3,1C and S8). For lay date in blue tits, the variance among forest clusters was five times higher than the variance among urban clusters; there was evidence that forest subpopulations differentiated to a greater extent in their mean lay dates compared to urban subpopulations (parameter 1 blue tits: urban < forest among‐cluster variance in means, Table 1C; Figures 3,1C and S8).

**TABLE 1:** Fixed and random effect estimates specified in the mean and dispersion (i.e., explains residual variation) parts of a double hierarchical linear model (DHGLM) when examining the effect of urbanisation (forest vs. urban) on the mean and residual variation of three traits: (A) adult tarsus length, (B) nestling tarsus length and (C) female lay dates.

	(A) Adult tarsus		(B) Nestling tarsus		(C) Lay date	
	Great tit *N* = 13,554	Blue tit *N* = 8414	Great tit *N* = 7505	Blue tit *N* = 2816	Great tit *N* = 6378	Blue tit *N* = 4419
**Mean part**						
*Fixed effects*						
Intercept (*β*_m0_)	0.208 [−0.3, 0.695]	0.23 [−0.645, 1.072]	−1.021 [−1.572, −0.458]	−0.716 [−1.72, 0.347]	0.256 [−0.011, 0.535]	0.293 [−0.24, 0.857]
Habitat (urban)	**−0.297 [−0.382, −0.216]**	**−0.182 [−0.272, −0.093]**	**−0.409 [−0.534, −0.284]**	−0.164 [−0.377, 0.037]	**−0.256 [−0.334, −0.176]**	−0.113 [−0.257, 0.029]
Latitude	**0.621 [0.282, 0.936]**	0.518 [−0.058, 1.075]	**0.518 [0.155, 0.832]**	0.596 [−0.19, 1.285]	**0.678 [0.488, 0.851]**	**0.726 [0.346, 1.054]**
(A) Sex (male) (B) Chick age	**0.541 [0.524, 0.559]**	**0.588 [0.564, 0.613]**	**0.085 [0.064, 0.105]**	**0.05 [0.018, 0.082]**		
*Random effects*						
Year	**0.038 [0.024, 0.057]**	**0.244 [0.189, 0.32]**	**0.112 [0.08, 0.156]**	**0.133 [0.091, 0.188]**	**0.395 [0.308, 0.506]**	**0.506 [0.39, 0.663]**
System	**0.89 [0.59, 1.379]**	**1.159 [0.696, 1.894]**	**0.755 [0.474, 1.207]**	**1.044 [0.607, 1.738]**	**0.387 [0.247, 0.623]**	**0.608 [0.337, 1.089]**
Cluster: (Parameter 1)						
Forest	**0.056 [0.033, 0.083]**	**0.039 [0.004, 0.094]**	**0.133 [0.082, 0.201]**	**0.335 [0.215, 0.5]**	**0.162 [0.122, 0.212]**	**0.327 [0.227, 0.458]**
Urban	**0.234 [0.168, 0.312]**	**0.144 [0.046, 0.264]**	**0.286 [0.2, 0.393]**	**0.149 [0.025, 0.3]**	**0.118 [0.07, 0.175]**	**0.063 [0.003, 0.173]**
**Dispersion part**						
*Fixed effects*						
Intercept (*βv*_0,exp_)	−0.545 [−0.699, −0.391]	−0.576 [−0.89, −0.213]	−0.434 [−0.624, −0.24]	−0.492 [−0.72, −0.266]	−0.734 [−0.845, −0.608]	−0.506 [−0.715, −0.299]
Habitat (urban; parameter 2)	0.04 [−0.016, 0.092]	**0.109 [0.008, 0.201]**	**0.109 [0.005, 0.215]**	**0.223 [0.057, 0.402]**	**0.086 [< 0.001, 0.174]**	0.093 [−0.03, 0.213]
Heterogeneity (1000 m)	−0.009 [−0.036, 0.017]	0.001 [−0.043, 0.05]	−0.001 [−0.04, 0.038]	−0.017 [−0.084, 0.055]	0.008 [−0.034, 0.049]	−0.008 [−0.068, 0.045]
Latitude	0.046 [−0.054, 0.154]	**0.29 [0.072, 0.533]**	0.098 [−0.029, 0.238]	0.085 [−0.078, 0.229]	−0.039 [−0.115, 0.038]	−0.036 [−0.192, 0.103]
Cluster area	**0.028 [0.011, 0.045]**	**0.161 [0.027, 0.285]**	0.01 [−0.024, 0.044]	0.177 [−0.066, 0.406]	−0.035 [−0.094, 0.023]	−0.024 [−0.17, 0.124]
Cluster years	0.002 [−0.049, 0.054]	−0.001 [−0.112, 0.101]	−0.003 [−0.085, 0.075]	−0.118 [−0.307, 0.07]	0.031 [−0.033, 0.101]	0.089 [−0.017, 0.182]
(A) Sex (male) (C) Age (1)	0.02 [−0.005, 0.043]	0.003 [−0.026, 0.035]			−0.03 [−0.072, 0.011]	−0.035 [−0.089, 0.019]
*Random effects*						
System (intercept)	**0.249 [0.151, 0.415]**	**0.334 [0.115, 0.731]**	**0.27 [0.154, 0.462]**	**0.082 [0.003, 0.273]**	**0.134 [0.061, 0.25]**	**0.203 [0.063, 0.488]**
System (*r*_mean,dispersion_)	−0.135 [−0.605, 0.377]	−0.157 [−0.701, 0.474]	−0.15 [−0.648, 0.407]	−0.046 [−0.79, 0.735]	0.376 [−0.223, 0.801]	0.301 [−0.417, 0.846]
Cluster: (Parameter 3)						
Forest (intercept)	**0.059 [0.027, 0.096]**	**0.043 [0.002, 0.109]**	**0.121 [0.077, 0.179]**	**0.205 [0.086, 0.359]**	**0.151 [0.104, 0.211]**	**0.067 [0.003, 0.177]**
Forest (*r*_mean,dispersion_)	−0.291 [−0.789, 0.31]	−0.122 [−0.846, 0.728]	**−0.491 [−0.834, −0.005]**	−0.452 [−0.877, 0.172]	−0.144 [−0.518, 0.267]	0.214 [−0.625, 0.804]
Urban (intercept)	**0.058 [0.005, 0.127]**	**0.109 [0.011, 0.251]**	**0.172 [0.112, 0.25]**	**0.142 [0.011, 0.304]**	**0.1 [0.013, 0.185]**	**0.161 [0.048, 0.306]**
Urban (*r*_mean,dispersion_)	−0.305 [−0.856, 0.477]	−0.013 [−0.733, 0.713]	**−0.767 [−0.97, −0.375]**	−0.259 [−0.874, 0.57]	−0.027 [−0.661, 0.609]	0.068 [−0.753, 0.808]

*Note:* Great and blue tit data were run in separate models (*N* = number of individuals/observations shown for each). Model estimates are shown with their 95% highest posterior density intervals (HPDIs); fixed effects and correlations in bold have HPDIs that exclude zero. Random effects with HPDI above 0.001 are in bold. Response and continuous variables were standardised before fitting models (mean = 0, standard deviation = 1), and effects in the dispersion part of the model are on the log scale. Back‐transformed fixed‐effect estimates are in Table S7.

### 3.2 Within‐Subpopulation Heterogeneity Hypothesis (H2 and Parameter 2)

For adult tarsus length, urbanisation increased the residual variance within clusters (parameter 2: positive habitat effect in Tables 1A and S4), with this effect having stronger evidence in blue tits (HPDI not overlapping zero) and weaker evidence in great tits (HPDI overlaps zero in great tits with pd = 0.93; similar results for ISA in Tables S5 and S6). The mean residual standard deviation for adult tarsus length in forest and urban great tits was estimated as 0.60 and 0.62 mm, respectively (a 4.1% increase in urban habitats; back‐transformed estimates from Tables 1A and S7; Figure 3,2A). The difference in variation between habitat types was higher in blue tits where the estimated mean residual standard deviation of adult tarsus length for forest and urban blue tits was estimated as 0.49 and 0.55 mm (an 11.5% increase in urban habitats; Figure 3,2A; Table S7). For nestling tarsus length, there was evidence that urbanisation increased the variation within clusters for both species (parameter 2: positive estimates of habitat effect with HPDIs excluding zero in Table 1B; and ISA in Tables S5 and S6). The mean residual standard deviation of nestling tarsus length for forest and urban great tits was estimated as 0.83 and 0.92 mm (an 11% increase in urban habitats; Figure 3,2B; Table S7). The difference in variation between habitat types was higher in blue tits where the estimated mean residual standard deviation of nestling tarsus length for forest and urban blue tits was 0.65 and 0.81 mm (a 25% increase in urban habitats; Figure 3,2B; Table S7). For lay date, urbanisation increased lay date variation within clusters for both species (parameter 2: positive urban habitat effect on residual variance in Table 1C; same for ISA in Tables S5 and S6), with this effect having stronger evidence in great tits than blue tits (weak evidence in blue tits with HPDI overlapping zero and pd = 0.94; Figure 3,2C). The mean residual standard deviations of lay date for forest and urban great tits were estimated as 5.6 and 6.1 days (8.9% increase in urban habitats; Tables 1C and S7), and 6.1 and 6.6 days for forest and urban blue tits (an 8.2% increase in urban habitats).

### 3.3 Heterogeneity in Heterogeneity Hypothesis (H3 and Parameter 3)

For all three traits in both species, estimates of among‐cluster variance in the residual variance did not differ between urban and forest habitat types; urban and forest clusters were similar in their variation in residual variance (parameter 3: urban ~ forest among‐cluster variance in residual variance with largely overlapping HPDIs; Table 1A–C; Figures 3,3A–C and S8).

## 4 Discussion

Using a mega‐analysis approach with long‐term data from 21 European urban and nonurban tit populations (13 great tit and 8 blue tit populations), we confirm strong phenotypic divergence in mean urban tarsus morphology and breeding phenology (see Supporting Information Results), and find some evidence that urbanisation can increase phenotypic variation at multiple levels. Urbanisation is associated with increased differentiation in tarsus length between urban subpopulations compared to forest subpopulations of great tits (i.e., clusters; H1: among‐subpopulation heterogeneity in Figures 1,1 and 3,1A,B), with weaker statistical support for this hypothesis in adult blue tits. This result, however, was lacking or even opposite when considering how urbanisation affected differentiation in lay date between subpopulations, with forest subpopulations of blue tits differentiating more than urban ones (Figure 3,1C). We also found statistical support in four out of the six species‐trait comparisons that urbanisation increases the phenotypic variation contained within subpopulations (H2: within‐subpopulation heterogeneity in Figures 1,2 and 3,2), a trend that was in the predicted positive direction across all comparisons, providing more general support that trait diversity exists at fine spatial scales in cities. We did not find support for higher differences in trait variation between urban subpopulations than forest ones (H3: heterogeneity in heterogeneity in Figures 1,3 and 3,3) suggesting that subpopulations in cities do not differ in their phenotypic variation more than subpopulations outside cities. We discuss results related to H1 and H2 in more detail below but, since we do not find statistical support for H3, we do not discuss these results in further detail.

Collectively, our results show that (i) phenotypic variation can be associated with urban conditions and processes at local scales within subpopulations and (ii) urbanisation might also increase differences between subpopulations at landscape scales (i.e., larger spatial scales) depending on the trait. In two of the trait‐species comparisons, we found evidence that urbanisation affects variation among and within subpopulations simultaneously, with these effects being in the same (nestling tarsus length in great tits: increases in variation at both levels) or opposite directions (lay date in blue tits: increases and decreases in variation at different levels). Despite numerous hypotheses on how ecological and evolutionary processes can affect phenotypic changes in urban populations (Alberti et al. 2020; Diamond and Martin 2021; Rivkin et al. 2019; Szulkin et al. 2020; Thompson et al. 2022), there are still limited empirical examples (Lambert et al. 2021). Although this synthesis does not directly evaluate the ecological and evolutionary processes acting in cities, it is a first step using a novel approach to generate hypotheses that may explain spatial patterns of urban phenotypic variation and consider the possible consequences of urban‐modified variation at different population levels (Box 1). These results highlight exciting avenues to determine whether higher individual heterogeneity in cities may be associated with, for example, higher evolutionary potential, more plastic responses to environmental variation, or differential dispersal in urban birds.

BOX 1 **Consequences of urban variation at different population levels.**

Beyond identifying plausible processes that shape variation in urban populations, we also discuss here the possible consequences of increased variation at different population levels (Bolnick et al. 2011; Mimura et al. 2017; Violle et al. 2012). At the within‐subpopulation level, fine‐scale phenotypic variation could give urban subpopulations the ability to respond to new or fluctuating selection pressures and buffer environmental variation and, if this phenotypic variation is underlined by similar patterns in genetic variation, the ability to adapt to further environmental change (Mimura et al. 2017; Moran et al. 2016). For example, the cues predicting optimal lay date timing could be less clear in urban environments (Schlaepfer et al. 2002) and so the fine‐scale variation in lay dates observed here could buffer unpredictable annual variation in optimal timing in urban environments. More diverse urban lay dates could also impact urban food webs by, for example, having top‐down selective consequences for the phenology of urban insect communities, with further consequences for phenology in urban tree communities (Jensen et al. 2022).

At the among‐subpopulation level, morphological variation in urban contexts could contribute to higher intraspecific diversity. In mammals, lower interpopulation variation in body size was correlated with a species' Red List status and vulnerability to extinction (González‐Suárez and Revilla 2013), which could suggest that higher tarsus length differences observed here between urban subpopulations could buffer the risks of local extinctions. Higher differences between urban subpopulations in the variation they contain compared to nonurban subpopulations (Hypothesis 3) could indicate that subpopulations in cities may vary more in their ability to adjust their phenotypic plasticity or adapt to further environmental change, but we did not find clear evidence here for this scenario. Evaluating dispersal between urban subpopulations will be particularly important for identifying further consequences at this population level. For example, if movements between subpopulations are limited within the urban matrix in less dispersive species, this could lead to further phenotypic (or genetic) differentiation in cities, with possible implications for adaptive radiations or speciation (Littleford‐Colquhoun et al. 2017; Thompson et al. 2018).

### 4.1 Hypothesis 1: Among‐Subpopulation Heterogeneity Hypothesis

In support of H1, we found higher differences in mean adult tarsus length between urban subpopulations of great tits than forest ones (adults and nestlings; Figure 3,1A,B). We found weaker evidence in adult blue tits for these higher urban subpopulation differences, and no clear support in blue tit nestlings (pattern in opposite direction; Figure 3,1A,B). For lay date, we did not find support for H1 as urbanisation was not associated with increased lay date differences between subpopulations in great tits, and there was opposite support for this hypothesis in blue tits (Figure 3,1C). Less consistent support for H1 may suggest that the processes that drive changes in phenotypic variation at large spatial scales in cities are less generalisable across the species and traits considered here.

Differentiation between urban subpopulations of great tits in tarsus length (and possibly adult blue tits) could be linked to heterogeneity in environmental conditions or dispersal between urban habitats. Differences between urban habitats in nestling food resources (and nutrients like carotenoids, Biard et al. 2006; Eeva et al. 2009; Isaksson 2009) might drive higher urban variation between subpopulations as it has been shown that food supplementation more strongly increases tarsus length in urban great tit nestlings than forest nestlings (Seress et al. 2020). Further, if urban great tits have limited dispersal after fledging, then phenotypic (and potentially genetic) differences between subpopulations reflect those seen in nestlings, especially since tarsus length is heritable and fixed early in life (*h* ^2^ = 0.3–0.8 in great tits, Young and Postma 2023). Further studies providing insights on the quantitative genetics and dispersal dynamics of urban bird populations (Hanmer et al. 2022; Senar and Björklund 2021) would help establish whether these patterns of tarsus length variation in adults could mirror genetic divergences in these tit populations.

We did not find support that urbanisation is associated with increased lay date differences between subpopulations of great tits, which suggests that environmental conditions or underlying genetic variation related to lay date may not vary between subpopulations at larger landscape scales in cities. In blue tits, we found opposite support for H1, with blue tit subpopulations differing more in their average lay dates in forests compared to urban areas (Figure 3,1C). This was a surprising result that has not been, to our knowledge, previously reported and so we are unable to offer a solid explanation for this pattern. Forests across Europe may be more diverse than cities in the environmental conditions that drive interannual differences in lay date timing (e.g., tree composition and phenology; Bailey et al. 2022). However, this would not fully explain why this result was unique to blue tits, although some studies have reported habitat‐dependent differences between these species in their lay date responses (Branston et al. 2021; Cuchot et al. 2024; Dhondt et al. 1984; Matthysen et al. 2021; Vaugoyeau et al. 2016). If great tits and blue tits differ in their perception of environmental cues (e.g., photoperiod or spring temperature), this could explain our results if processes like light pollution or the urban heat island effect homogenise these cues at larger spatial scales in cities (Branston et al. 2021; Yao et al. 2018). While these explanations are speculative, urbanisation clearly alters the phenological distribution (mean and width) of urban tit populations. Given the multitude of ramifications that can be triggered by the interactive effects of climate change and the urban heat island (Urban et al. 2024), it will be important to explore these patterns further to establish how urban and forest tits are differentially responding to advances in spring phenology under a warming climate.

### 4.2 Hypothesis 2: Within‐Subpopulation Heterogeneity Hypothesis

We found consistent support for H2 across the six species‐trait combinations, with urbanisation increasing phenotypic variation within subpopulations by 11% on average (range: 4.5%–25%; Figure 3,2). More specifically, urbanisation was associated with an 8.5% increase in lay date variation. This effect is weaker than the average effect of urbanisation on lay date variation reported across 34 bird species globally (19.2% increase; Capilla‐Lasheras et al. 2022). In contrast, the increase in variation that we report here is stronger than reported in a recent meta‐analysis showing that human disturbances outside urban contexts (e.g., climate change, pollution, harvesting) have negligible effects on phenotypic variation, with most wild populations showing changes of only ~1% in variation on average (Sanderson et al. 2023). It is unclear whether these urban tit populations represent an extreme case of how human disturbance impacts variation, like a few cases reported in Sanderson et al. or whether disturbance in urban environments can have similar impacts on phenotypic variation as manipulated stressors in experiments (O'Dea et al. 2019; Sánchez‐Tójar et al. 2020). Further mega‐ or meta‐analyses that include studies from multiple taxa (especially outside birds) and traits in urban environments will be needed to further generalise how ecological conditions related to urbanisation impact phenotypic variation in the wild.

We found evidence that urbanisation increased tarsus length variation within subpopulations across both species and life stages examined (predicted positive direction; Figure 3,2A,B), aside from adult great tits where evidence was weaker. Higher tarsus diversity within urban subpopulations could be driven by similar processes discussed above for H1 acting at finer spatial scales within urban habitats. For example, fine‐scale tarsus length diversity could be driven by limited or non‐random dispersal within an urban habitat related to the quality of breeding territories. Variation in body mass, for instance, is spatially variable in a woodland population of great tits, and this fine‐scale phenotypic diversity may be primarily driven and maintained by non‐random dispersal related to habitat quality (Garant et al. 2005). If individuals recruit locally, this could drive a process where more diverse urban nestlings lead to diverse adults within a subpopulation.

The estimated effect of urbanisation on tarsus length variation within subpopulations was larger and had stronger evidence in nestlings than adults. Note that we use direct urbanisation data related to the environment that nestling tarsus developed in, whereas adults were measured in the environment they chose after dispersing, which could create noise around urbanisation's effect on adult tarsus length. However, selective processes that cause non‐random disappearance from the population between the nestling and adult stages (e.g., lighter nestlings less likely to fledge; Corsini et al. 2021) could also explain less tarsus length variation observed in urban adults. Individual‐based demographic models would be useful to evaluate how certain phenotypes (e.g., being smaller or larger than average) affect survival probabilities after fledging in urban and nonurban populations (DeAngelis and Grimm 2014; Dunlop et al. 2007).

Within subpopulations, we found evidence that urban females tend to have more diverse lay dates than forest females, suggesting that previously reported increased lay date variation in urban bird populations (Capilla‐Lasheras et al. 2022) could arise at local spatial scales in cities (evidence in great tits and weak evidence in blue tits; Figure 3,2C). Increased lay date diversity within urban areas could be driven by variation among urban females in (i) lay date plasticity to fine‐scale heterogeneity in urban environmental conditions (e.g., temperature, artificial light at night, vegetation cover, tree species and their phenology; Bailey et al. 2022; Bonamour et al. 2019; Dominoni et al. 2020; Jensen et al. 2022; Matthysen et al. 2021; Monniez et al. 2022; Shutt et al. 2019) or (ii) lay date timing (and associated genetic variation) which might result from relaxed urban selection on breeding phenology (as shown in great and blue tits: Branston et al. 2021; Caizergues et al. 2018). Additional data collection across these urban tit populations would allow a more ambitious approach that incorporates pedigrees in our models, allowing an evaluation of whether genetic variation of lay date (or tarsus length) underlies these patterns of phenotypic variation at these population levels.

### 4.3 Limitations and Moving Forward

Our synthesis takes advantage of a large collaborative effort that is pushing to standardise research approaches and combine long‐term data (SPI‐Birds; Culina et al. 2020) and, although these large collaborative efforts will be key for establishing broad conclusions across replicate populations or cities, there are relevant limitations here that warrant discussion. Our data include some long‐term studies on great and blue tits that were initially studied in forest habitats. Therefore, it is unclear whether differences in phenotypic variation reported here between urban and forest populations also extend to how variation differs between urban and nonurban (e.g., grassland or marsh) populations more generally. Further, our dataset comprises more replicate populations that exist across a larger geographical range for great tits than blue tits, and so population‐level patterns that differ between the species here should be interpreted with this in mind.

We found no clear associations between large‐scale land cover heterogeneity and phenotypic variation across the traits and species examined. This null result may be due to the cruder resolution of the Europe‐wide land cover data we used (1 × 1 km classification as opposed to 10 m resolution of impervious surface area), rather than a true absence of an effect. Environmental heterogeneity can occur at fine spatial scales (within meters) and could be more likely to drive the fine‐scale trait variation we observed here since our clusters contained nest boxes that were within 300 m. Studies measuring fine‐scale environmental data will be needed to explore further how environmental variation translates into phenotypic variation. Establishing this link will be especially meaningful in urban habitats since they are assumed to be more environmentally heterogenous, but this assumption may depend on the environmental axis and scale considered (Thompson et al. 2022). Accumulating data on different axes of environmental variation, such as temperature, vegetation cover or artificial light at night and examining how these factors differentially impact traits would allow a more comprehensive examination of how urbanisation impacts variation at local scales.

It is also important to reiterate that our estimates of trait variation do not distinguish among‐ and within‐individual variation, or importantly underlying genetic and environmental components of this variation. Although our approach constitutes an initial synthesis of how urbanisation modifies individual diversity across the continent, further efforts will be needed to decipher the underlying components of this variation. More complex approaches including double hierarchical animal models (e.g., Martin et al. 2017) or fine‐scale genomic‐based population analyses (e.g., Whitaker et al. 2020) could be useful complements to this end. Future studies that aim to examine similar hypotheses in different urban systems should importantly consider how best to define a subpopulation. We outline how the spatial scale used to define subpopulations could impact how variation is captured among and within subpopulations in Box 2.

BOX 2 **Considerations when defining subpopulations.**

Subpopulations in wild systems are generally defined as being genetically differentiated groups of individuals within a larger population (Verity and Nichols 2016). However, information on the genetic population structure of most wild systems is not always available, especially in cases where several replicate populations are studied at broad spatial scales. Therefore, researchers rely on defining subpopulations by grouping observed individuals in space, but should be aware that the spatial scale used to define subpopulations could impact how phenotypic variation is captured among and within subpopulations. For example, if subpopulations are defined to be larger in space, then the processes that drive phenotypic differences between subpopulations (H1 and H3) could be captured by increasing the amount of phenotypic variation within populations (H2). Conversely, if subpopulations are defined too small, then the processes that increase phenotypic variation within populations (H2) could contribute to phenotypic differences between populations (H3). Individuals in wild populations are rarely studied intensively in space and so study designs that involve the monitoring of individuals across spatially separated study sites (like tits monitored here in, for example, forest patches or urban green spaces) will have limited capacity to explore how altering the spatial extent of subpopulations could change conclusions. Nonetheless, this is a caveat that should be considered by researchers wishing to address similar hypotheses and we choose here to define subpopulations using biologically relevant spatial scales linked to the space use of tits during breeding (see Supporting Information Methods Section 1.5).

## 5 Conclusions

Urbanisation is associated with increases in variation within subpopulations, although the strength and statistical evidence for this pattern depend on the species and trait examined. This suggests that processes like selection, dispersal or plasticity affect phenotypes at fine spatial scales in cities which, in turn, have consequences at different population levels (Box 1). Urbanisation can also impact variation at multiple population levels simultaneously; we found clear statistical support for nestling great tit tarsus lengths (urbanisation increases variation at both levels) and blue tit lay dates (urbanisation has opposing effects at the different levels). Large collaborative efforts will be powerful approaches for synthesising more generally how urbanisation is impacting wildlife as these large datasets open new research possibilities and the ability to draw broad insights across replicate cities. Single system research using experiments or finer scale data will be necessary complements to these mega‐analyses, and together single and multi‐population approaches can make timely fundamental and applied contributions in urban ecology and evolution. Determining how ecological conditions like urbanisation affect phenotypic variation is an especially significant research avenue to establish the programmes needed to conserve wild populations and ecological communities in cities (Mimura et al. 2017), so that nature's contributions to society (Des Roches et al. 2021) are maintained in the face of the Anthropocene.

## Author Contributions

M.J.T., J.G.A.M., D.R. and A.C. conceived the study. M.J.T., C.B., J.B., C.J.B., P.C.‐L., N.J.D., D.M.D., M.E., T.E., K.L.E., C.I., A.L., S.M., E.M., A.M., S.P., J.C.S., G.S., M.S., E.V., H.W. and A.C. developed the study methodology, and collected and prepared data. M.J.T. compiled the data, performed the analyses and wrote the first draft of the manuscript. J.G.A.M. advised on the analyses and M.J.T., J.G.A.M., D.R. and A.C. interpreted the results. All authors contributed substantially to reviewing and editing drafts of the manuscript.

## Peer Review

The peer review history for this article is available at https://www.webofscience.com/api/gateway/wos/peer‐review/10.1111/ele.70180.

## Supporting information


Data S1.

## Data Availability

All data and code to reproduce this study's results are available on the Dryad repository at https://doi.org/10.5061/dryad.r2280gbns.
