# Supplementary material for: Continental Patterns of Phenotypic Variation Along Replicated Urban Gradients: A Mega‐Analysis
Source: Ecol Lett. 2025 Jul 24;28(7):e70180. doi: 10.1111/ele.70180 (PMC12288444; doi:10.1111/ele.70180)
Supplement: Supplementary file 1 — Data S1. [file ELE-28-0-s001.docx]

**Supporting Information**

**Title:**

Continental patterns of phenotypic variation along replicated urban gradients: a mega-analysis

**Author Names:**

Thompson, M.J._1,2_, Martin, J.G.A._3_, Biard, C._4_, Bleu, J._5_, Branston, C. J._6,7_, Capilla-Lasheras, P._6,8,9_, Dingemanse, N.J._10_, Dominoni, D.M._6_, Eens, M._11_, Eeva, T._12_, Evans, K.L._13_, Isaksson, C._14_, Liker, A._15,16_, Massemin, S._5_, Matthysen, E._11_, Mouchet, A._10_, Perret, S._2_, Senar, J.C._17_, Seress, G._15_, Szulkin, M._18_, Vincze, E._14,15_, Watson, H._14_, Réale, D._1_, Charmantier, A_2_.

**Affiliations:**

_1_Département des sciences biologiques, Université du Québec à Montréal, 141 Avenue du Président-Kennedy, Montréal, QC H2X 1Y4, Canada

_2_Centre d’Ecologie Fonctionnelle et Evolutive, Univ Montpellier, CNRS, EPHE, IRD, Montpellier, France

_3_Department of Biology, University of Ottawa, 30 Marie-Curie Private, Ottawa, Ontario K1N 9B4, Canada

_4_Sorbonne Université, Université Paris Cité, Univ Paris Est Créteil, CNRS, IRD, INRAE, Institut d'écologie et des sciences de l'environnement de Paris, IEES, F-75005 Paris, France

_5_Université de Strasbourg, CNRS, Institut Pluridisciplinaire Hubert Curien, IPHC UMR 7178, 67000 Strasbourg, France

_6_School of Biodiversity, One Health and Veterinary Medicine, University of Glasgow, Glasgow, UK

_7_School of Health and Life Sciences, University of the West of Scotland, Lanarkshire, UK

_8_Swiss Ornithological Institute, Sempach, 6204, Switzerland

_9_Doñana Biological Station (CSIC), Seville 41092, Spain

_10_Behavioural Ecology, Faculty of Biology, LMU Munich, 82152 Planegg-Martinsried, Germany

_11_Department of Biology, University of Antwerp, Wilrijk, Belgium

_12_Department of Biology, University of Turku, Turku 20014, Finland

_13_Ecology and Evolutionary Biology, School of Biosciences, University of Sheffield, Sheffield S10 2TN, United Kingdom

_14_Department of Biology, Lund University, Lund, SE-223 62 Sweden

_15_HUN-REN-PE Evolutionary Ecology Research Group, University of Pannonia, Veszprém H-8200, Hungary

_16_Behavioral Ecology Research Group, Center for Natural Sciences, University of Pannonia, Veszprém H-8200, Hungary

_17_Departament of Evolutionary and Behavioural Ecology, Museu de Ciències Naturals de Barcelona, Barcelona 08003, Spain

_18_ Institute of Evolutionary Biology, Faculty of Biology, Biological and Chemical Research Centre, University of Warsaw, Warsaw, Poland

**Corresponding author:**

Megan J. Thompson (thompsonjoymegan@gmail.com)

**Supporting Information Contents**

**Section 1. Supplementary methods**

*1.1. Conversion to Svensson’s Alternative method (equations S1-2)*

*1.2. Mean adult tarsus length observation*

*1.3. Random nestling tarsus length observation*

*1.4. First lay date observation*

*1.5. Environmental variables*

**Section 2. Supplementary results**

*2.1. Urbanization drives divergence in mean tarsus length and lay date*

*2.2. Supplementary results on adult tarsus length*

*2.3. Supplementary results on nestling tarsus length*

*2.4. Supplementary results on lay date*

**Section 3. Figures**

*S1-S3. Number of individuals for each cluster across study systems, species, and traits*

*S4. Proportion impervious surface area in urban and forest habitats across study systems*

*S5. Relationship between impervious surface area and land cover diversity*

*S6. The effect of urban vs forest habitat on trait means*

*S7. The effect of impervious surface area on trait means*

*S8. Difference between urban and forest posterior draws in variance*

**Section 4. Tables**

*S1. Summary of datasets across study systems*

*S2. Summary of urban vs forest cluster characteristics across study systems*

*S3. Re-analysis of adult tarsus length using random observation per individual*

*S4. Summary of clustering algorithm at different spatial scales*

*S5. Re-analysis when using proportion ISA at 1000m scale instead of habitat type*

*S6. Re-analysis when using proportion ISA at 100m scale instead of habitat type*

*S7. Back-transformed fixed-effect estimates from Table 1*

*S8. Re-analysis when excluding Munich city habitat cluster*

*S9. Model equation terms and notation*

**Section 5. Supplementary model equations for adult tarsus length**

*Equations S3-8*

**Section 1. Supplementary methods**

*1.1. Conversion to Svensson’s Alternative method*

We used individual tarsus length measurements using the Svensson’s Alternative Method (Svensson 1992), which is measured from the intertarsal notch to the end of the tarsus by bending the foot. For two study systems, we converted tarsus length measurements using the Svensson’s Standard or Oxford Maximum methods (see Table S1) to the Svensson’s Alternative method using the established equations from SPI-Birds (Culina *et al.* 2020a, b):

| $Svensson^{'}s alternative=Svensson^{'}s standard \times0.777 +6.158$ | [Eq.S1] |
| --- | --- |
| $Svensson^{'}s alternative=Oxford maximum \times0.72005 +6.158$ | [Eq.S2] |

*1.2. Mean adult tarsus length observation*

Adults could be caught and measured multiple times if they bred in multiple breeding seasons. For adult tarsus, an individual’s tarsus length was measured 1.4 times on average across studies (great tit mean = 1.5, range = 1 – 9; blue tit mean = 1.45, range = 1 – 7). We decided to take the mean tarsus value for each individual since tarsus length is fixed early in life and should not change over subsequent measures; taking the mean of this trait should reflect an individual’s size over their life while reducing measurement error. Note that the number of adult tarsus measurements per individual was similar across habitats for both species (urban great tit: mean = 1.4, range = 1 – 7, forest great tit: mean = 1.6, range = 1 – 9, urban blue tit: mean = 1.3, range = 1 – 7, forest blue tit: mean = 1.6, range = 1 – 7) and, our results were qualitatively similar when instead selecting a random observation per individual (Table S4), demonstrating low error across individual measurements (Pearson r^2^ = 0.99 between mean and randomly selected tarsus measures, N = 19366).

*1.3. Random nestling tarsus length observation*

We chose to take a random nestling observation per brood. We avoided taking the mean nestling tarsus measure per brood as we did not want to reduce the influence of extreme values (small or large nestlings) on the variation observed in nestling tarsus length. Although urban tit nestlings reach their full asymptotic growth later than forest nestlings (Corsini *et al.* 2021), we did not instead take the largest nestling observation per brood since i) this could also reduce biologically interesting differences in variation between urban and nonurban habitats at this life stage and ii) slower urban mean growth rate would not clearly bias the variation measured in tarsus length.

*1.4. First lay date observation*

An individual’s lay date was recorded 1.45 times on average (great tit mean = 1.5, range = 1 – 6; blue tit mean = 1.4, range = 1 – 6). Since we included the first observation of each females lay date, the number of lay date observations was higher for yearlings than older birds in both species (great tits: N = 4503 yearlings vs. 1875 older, blue tits: 3337 yearlings vs. 1082 older), with this ratio being higher in forest habitats (great tits: 1.8x more yearlings in urban vs. 2.7x in forest; blue tits: 2.1x more yearlings in urban vs. 3.5x in forest). We chose to select an individual’s first observation (73% of observations were yearlings), rather than their mean or a random observation, i) to avoid averaging a plastic trait across breeding attempts and ii) since female lifespan varies and can have impacts on the mean and variation of lay date (Auld & Charmantier 2011; Dhondt 1989).

*1.5. Environmental variables*

We generated clusters (i.e., subpopulations along urban gradients) using a DBSCAN clustering algorithm in QGIS (v3.22.0; QGIS Development Team 2023) that grouped together at least 5 nest boxes within a 300-meter distance of each other. Often, this definition grouped together nest boxes in a similar habitat and led to the same study areas as those defined by data owners (119 study areas; Table S1). Separation between clusters of at least 300 meters is biologically relevant as this tends to be beyond the documented foraging area of radio-tracked great and blue tits during the breeding season (i.e., Jarrett *et al.* 2020; Seress *et al.* 2025; Tremblay *et al.* 2005), and clusters of nest boxes gave similar results when clustering nest boxes within 200 and 400 meters (range = 136 – 106 clusters; Table S4). In the case of Munich, urban nest boxes did not cluster together because nest boxes throughout the city were on average 904 meters apart. Therefore, we manually added these nest boxes as one cluster and compared results when including and excluding this cluster. As clusters differed in their size and we wanted to control for the possibility that a cluster’s size could impact the variation they contained, we calculated cluster area (m^2^) as the total area covered by nest boxes using Minimum Bounding Convex Hulls in QGIS.

We quantified the degree of urbanization at each nest box (N = 7210) in the dataset. The biologically relevant scale at which we should quantify urbanization is rarely obvious, yet it is a crucial methodological choice (Uchida *et al.* 2021). For example, small- and large-scale urbanization quantified around a group of individuals occupying a small urban green space could be considerably different and may have varied effects on phenotypes depending on the species or trait under study (e.g., Kaiser *et al.* 2016; Strubbe *et al.* 2020; Waterschoot *et al.* 2023). We therefore chose to examine the effect of urbanization at both small and large spatial scales (100 vs. 1000 meters) relative to the species’ home range around the nest box (approx. 60 – 160 meters; Jarrett *et al.* 2020; van Overveld *et al.* 2011; Seress *et al.* 2025; Wilkin *et al.* 2006) and natal dispersal distance (up to 900 meters on average in females, Dingemanse *et al.* 2002; Garant *et al.* 2005; Szulkin & Sheldon 2008). We calculated the proportion of impervious surface area (ISA), defined as sealed non-natural surfaces (e.g., roads, railway tracks, buildings), as a metric for urbanization using the imperviousness density raster datasets from the Copernicus online database (resolution 10m; see European Environment Agency 2020). Using 100- and 1000-meter circular radius buffers around each nest box, we calculated the proportion of ISA by counting the number of pixels associated with imperviousness and divided this by the number of pixels within each buffer (range: 0 – 1, where 1 = all ISA). The proportion of ISA was highly correlated with the proportion of impervious built-up area across all nest boxes in our dataset (rho = 0.92 and 0.97 for 100- and 1000-m scales, respectively), and so we chose to only use ISA measures moving forward. When considering all nest boxes together, the quantified ISA at 100- and 1000-meter scales were correlated (rho = 0.75). To generate an ISA score for each cluster, we averaged the proportion ISA across all nestboxes within a cluster. Urban clusters were within or close to city centers and had higher values of proportion ISA (cluster mean = 0.46 proportion ISA at 100m, range = 0.002 – 0.98), whereas forest clusters were in forested areas outside cities and had lower proportion ISA (cluster mean = 0.01 proportion ISA at 100m, range = 0 – 0.19, see also Table S2 and Figure S4).

We also extracted large-scale land cover heterogeneity around each nest box using the CORINE Land Cover dataset that includes 44 different classes of land cover types (resolution 100m; European Environment Agency 2021). We calculated the number of pixels associated with each land cover type within 100- and 1000-m buffers around each nest box. Using these values, we calculated land cover diversity using the Shannon Diversity Index that accounts for both richness (total number) and evenness (relative abundance) of land cover types at each nest box. To generate land cover diversity at the cluster level, we again averaged diversity values across all the nest boxes within a given cluster. In our dataset, ISA had a negative quadratic relationship with land cover heterogeneity at both the nest box and cluster levels suggesting that moderately urbanized clusters (between 0.2 – 0.6 proportion ISA) tended to have higher landscape heterogeneity (Figure S5). All spatial datasets were projected in the Lambert azimuthal equal-area European projection (EPSG: 3035) because impervious surface area and land cover heterogeneity were estimated and compared across Europe. The associated raster tiles used for impervious density for each study system are listed in Table S1. See Table S2 for an overview of urban and forest cluster characteristics across each study system.

**Section 2. Supplementary results**

*2.1. Urbanization drives divergence in mean tarsus length and lay date*

Our synthesis confirms previous findings from single system studies (e.g., Biard *et al.* 2017; Caizergues *et al.* 2018; Corsini *et al.* 2021; Seress *et al.* 2020) on how urbanization is associated with smaller mean tarsus length and earlier lay date in tit species, with results being consistent when examining the effect of urbanization as categorical (urban vs. forest) or continuous (ISA gradient). Urban adult tits had shorter mean tarsus lengths than nonurban ones (20.13 and 19.82 mm for forest and urban great tits, 17.20 and 17.05 mm for forest and urban blue tits; Table 1A&S7; Figure S6) and tarsus length decreased with increasing proportion ISA (Table S5-6; Figure S7). Nestlings in urban habitat types had smaller tarsi, but this effect was weaker in blue tits (HPDI overlaps zero but posterior direction (pd) = 0.94; 18.31 and 17.79 mm in forest and urban great tits, 16.3 and 16.1 mm in forest and urban blue tits; Table 1B&S7; Figure S6). The effect of proportion ISA on nestling tarsus length, however, was clear in both species with nestlings in more urbanized habitats having smaller tarsi than those in less urbanized ones (Table S5-6; Figure S7). Urbanization (categorical and ISA) had a clear effect on mean lay date in great tits, but its effect in blue tits was weaker (note HPDI of habitat effect crossing zero for blue tits in Table 1C and pd = 0.94, see also ISA effects in Table S5-6). The average female lay date for forest and urban great tits was 109 and 106 Julian days (April 19 and 16), respectively, and forest and urban blue tit females had average lay dates of 105 and 103 Julian days (April 15 and 13; Table 1C; Table S5-7; Figure S6&S7). Although these results confirm that urbanization is associated with decreases in trait means in tits across Europe more generally, it is important to note that our general approach aimed at evaluating changes in trait variance, hence the modelling approach was not developed specifically to study differences in mean traits. For example, for simplicity we have not modeled changes in trait means across proportion ISA separately for each gradient (i.e., ISA as random slopes across Study System) and ignored possible non-linear changes in trait means. For these reasons, our results broadly synthesize how urbanization associates with trait means at a European level, but we do not investigate more complex patterns in mean traits here.

*2.2. Supplementary results on adult tarsus length*

In the mean part of the DHGLM model for adult tarsus length described in Table 1A, sex had an effect on mean tarsus length in both species with males having longer tarsi than females. Tits at higher latitudes had longer tarsi than tits at lower latitudes, with this effect being weaker in blue tits (HPDI overlaps zero but pd = 0.96; Table 1A). The variance among study systems in mean tarsus length was high for both species and these study system differences explained at least four times more variation than that among years (Table 1A).

Clusters with larger areas (both species) and higher latitude (blue tits) were associated with higher variation in tarsus length (Table 1A), while all other considered effects did not clearly affect tarsus length variation (HPDIs overlap zero; Table 1A). The mean residual standard deviation of adult tarsus length also varied across study systems (Table 1A). We found negative correlations between the mean and standard deviations of tarsus length across study systems and clusters (i.e., negative estimates for *r*_mean, dispersion_ of random effects in dispersion part of Table 1A) suggesting that systems and clusters that tend to contain individuals with smaller tarsi (lower mean) also contain individuals with more variable tarsus lengths (higher standard deviation). However, there was high uncertainty around these negative correlations (i.e., large HPDIs overlap zero) suggesting the relationship between the mean and variation of tarsus length either i) differed considerably among systems and clusters in this dataset or ii) we lacked power to estimate these correlations across clusters. Excluding the Munich cluster in a subsequent analysis did not change our main conclusions (Table S8A).

*2.3. Supplementary results on nestling tarsus length*

In the mean part of the DHGLM model for nestling tarsus described in Table 1B, the age of chicks had an effect on mean tarsus length in both species with older individuals having longer tarsi than younger ones. Similar to results on adult tarsus, nestlings at higher latitudes had longer tarsi than tits at lower latitudes, with again this effect being weaker in blue tits (HPDI overlaps zero but pd = 0.94; Table 1A). Similarly to adult tarsus length, the estimated variance in mean nestling tarsus length among study systems was high for both species and these between study system differences explained at least six times more variation than the variation among years (Table 1A).

In the dispersion part of the model, there were no clear associations between the fixed effects (heterogeneity, latitude, cluster area, and years) and tarsus length variation. There was high uncertainty around the estimated correlations between the means and residual standard deviations of nestling tarsus across study systems (i.e., large HPDIs overlapping zero for system *r*_mean, dispersion_ effect). In great tits, urban and forest clusters had clear negative correlations between the mean and residual standard deviations of tarsus size indicating that clusters that tended to contain smaller great tit nestlings also tended to contain more variable individuals (i.e., strong negative Cluster *r*_mean, dispersion_ estimates). These strong negative correlations in great tits were robust when running the model with a correlation prior with higher degrees of freedom (df = 5, leading to a stronger prior for an absence of a correlation). These cluster-level negative correlations were weaker in blue tits in forest clusters (HPDI overlaps zero but pd = 0.93) and not statistically supported in urban clusters (pd < 0.90). Excluding the Munich cluster in a subsequent analysis did not change our conclusions, and the effect of urbanization had a stronger effect on both the mean and residual variation of nestling tarsus in these models (Table S8B).

*2.4. Supplementary results on lay date*

In the mean part of the DHGLM model for lay date described in Table 1C, latitude clearly associated with mean lay date in both species where lay dates were later at higher latitudes (Table 1C). The estimated variance among study systems in mean lay date was high but, unlike the results for tarsus, the estimated variance among years was also high in both species (similar estimates of among-system and among-year variance in Table 1C).

In the dispersion part of the model, there were no clear associations between the fixed effects (heterogeneity, latitude, cluster area, years, and age) and lay date variation. Estimated correlations between the mean and standard deviations of lay date across study systems and clusters (i.e., estimates for *r*_mean, dispersion_ in Table 1A) were weak and highly uncertain.

**Section 3: Supplementary Figures**
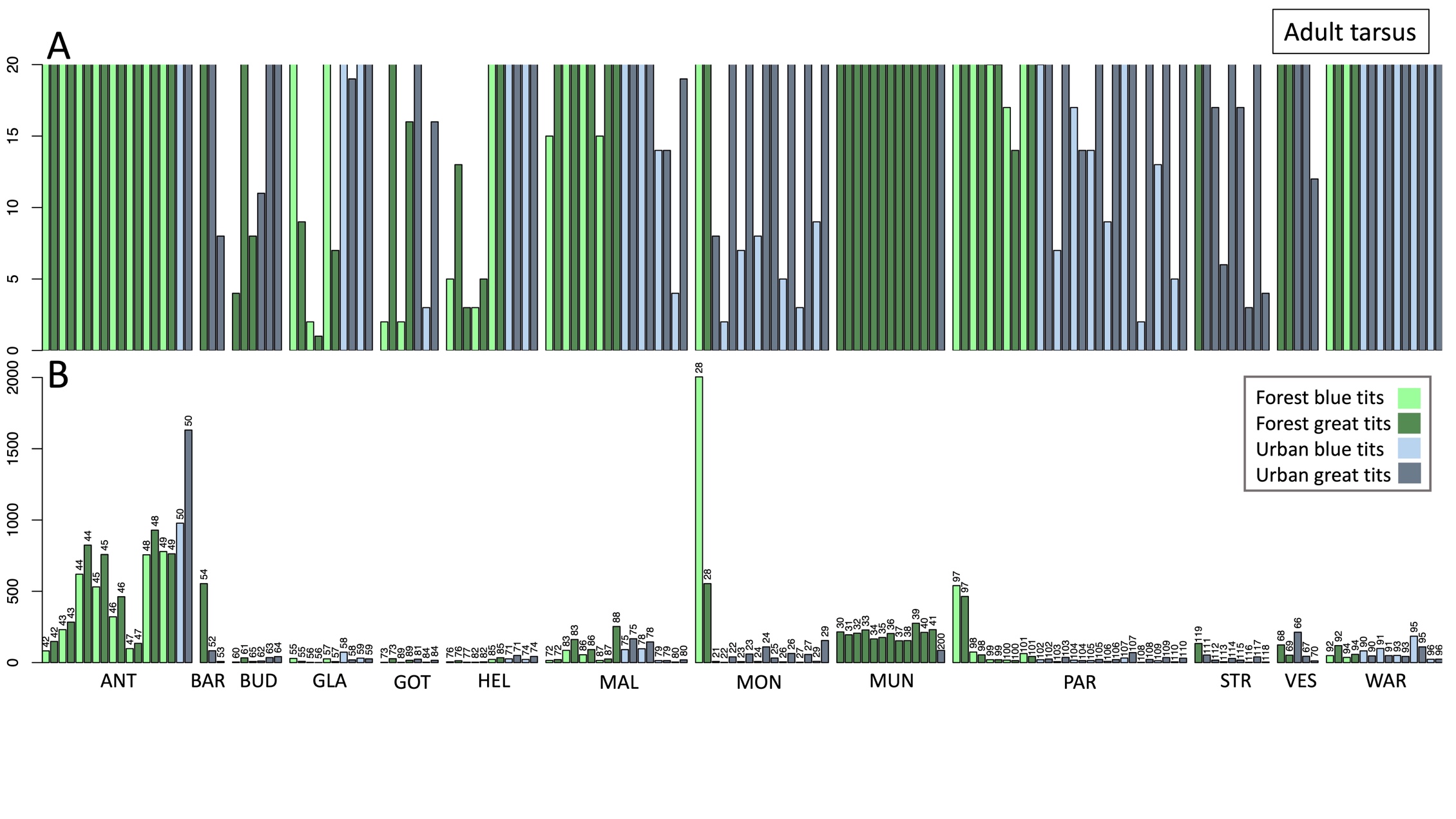


**Figure S1.** Number of individuals for adult tarsus length for each cluster (or groups of individuals) across study systems (ANT = Antwerp, BUD = Budapest, GLA = Glasgow, GOT = Göteborg, HAR = Harjavalta, HEL = Helsingborg, Malmo = Malmö, MON = Montpellier, MUN = Munich, PAR = Paris, STR = Strasbourg, VES = Veszprém, WAR = Warsaw). A) Top panel shows number of observations when range is constrained between 0 and 20 to better visualize instances of small sample size, where bars reaching maximum have 20 or more observations. B) Bottom panel expands top panel and shows full range of observations. Habitat type (forest = green, urban = blue), species (light colour = blue tit, dark colour = great tit), and the number related to each cluster ID (i.e., number above each bar in bottom panel; see also cluster ID numbers listed in Table S1) are shown.


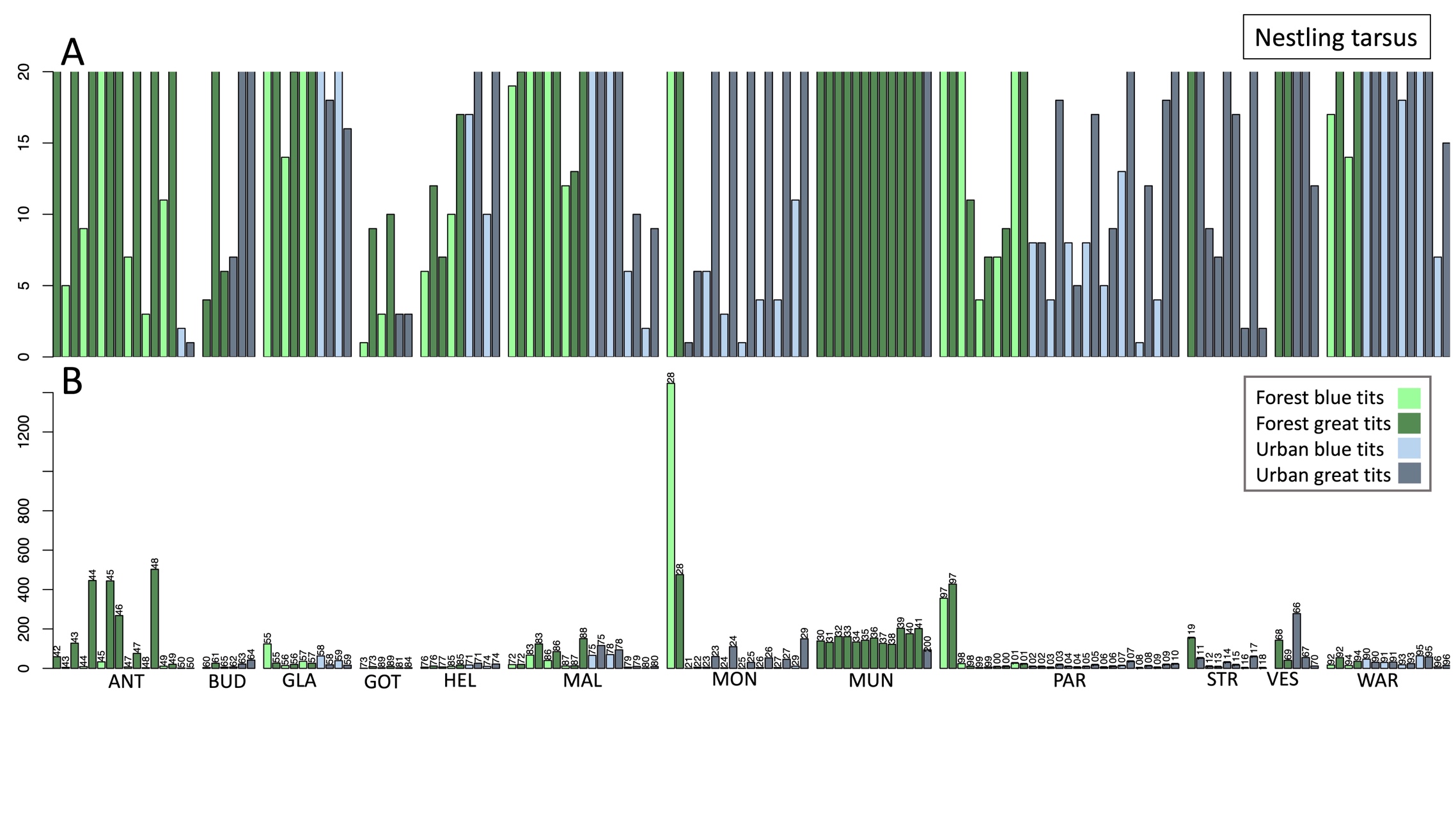


**Figure S2.** Number of individuals for nestling tarsus length for each cluster (or groups of individuals) across study systems (ANT = Antwerp, BUD = Budapest, GLA = Glasgow, GOT = Göteborg, HAR = Harjavalta, HEL = Helsingborg, Malmo = Malmö, MON = Montpellier, MUN = Munich, PAR = Paris, STR = Strasbourg, VES = Veszprém, WAR = Warsaw). A) Top panel shows number of observations when range is constrained between 0 and 20 to better visualize instances of small sample size, where bars reaching maximum have 20 or more observations. B) Bottom panel expands top panel and shows full range of observations. Habitat type (forest = green, urban = blue), species (light colour = blue tit, dark colour = great tit), and the number related to each cluster ID (i.e., number above each bar in bottom panel; see also cluster ID numbers listed in Table S1) are shown.


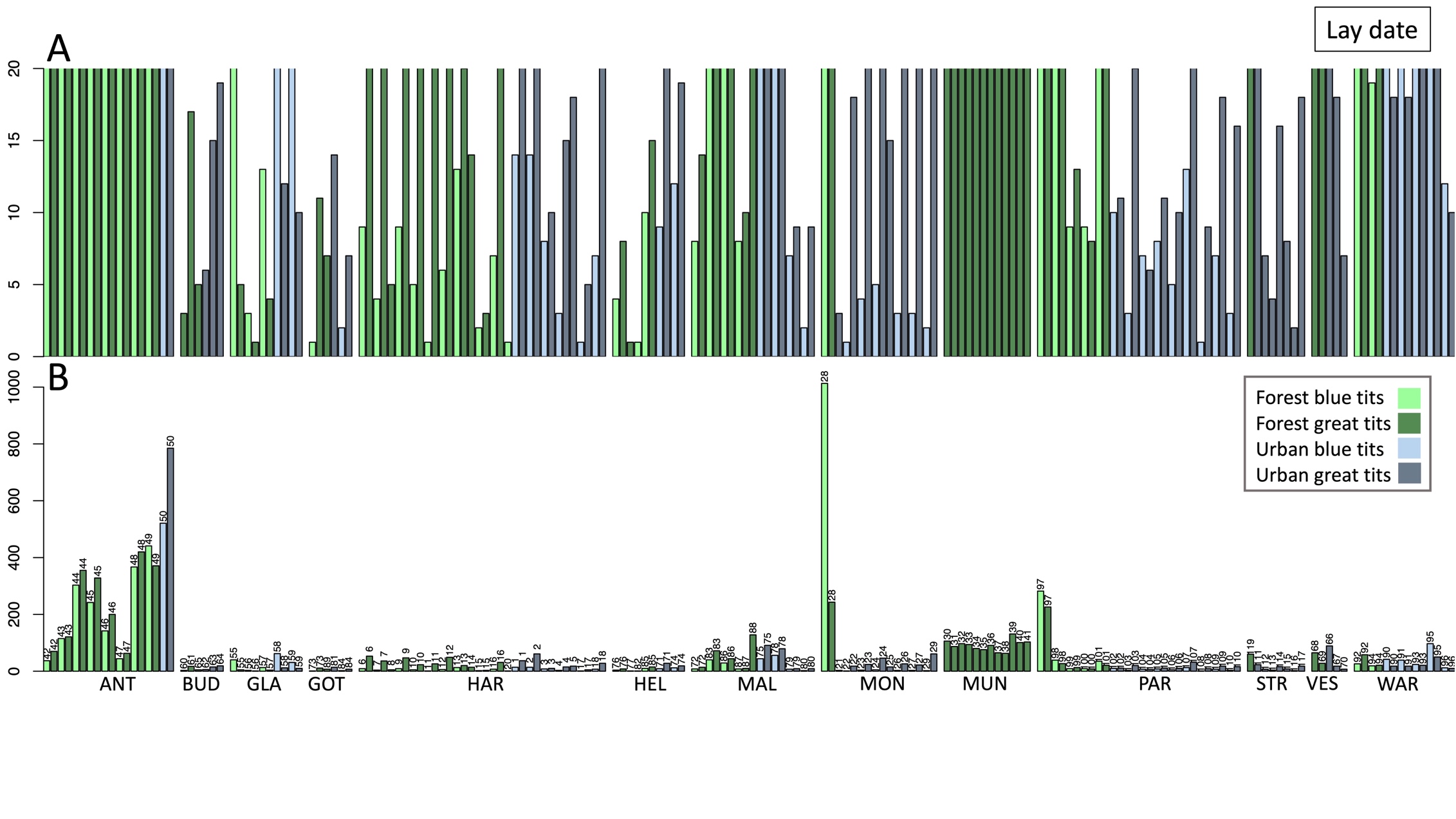


**Figure S3.** Number of individuals for female lay date for each cluster (or groups of individuals) across study systems (ANT = Antwerp, BUD = Budapest, GLA = Glasgow, GOT = Göteborg, HAR = Harjavalta, HEL = Helsingborg, Malmo = Malmö, MON = Montpellier, MUN = Munich, PAR = Paris, STR = Strasbourg, VES = Veszprém, WAR = Warsaw). A) Top panel shows number of observations when range is constrained between 0 and 20 to better visualize instances of small sample size, where bars reaching maximum have 20 or more observations. B) Bottom panel expands top panel and shows full range of observations. Habitat type (forest = green, urban = blue), species (light colour = blue tit, dark colour = great tit), and the number related to each cluster ID (i.e., number above each bar in bottom panel; see also cluster ID numbers listed in Table S1) are shown.

**Figure S4.** Boxplots (box = 25 – 75^th^ percentile, thick line = median) showing the proportion impervious surface area (ISA) quantified around each nest box within radius circular buffers of A) 1000 meters and B) 100 meters in urban (blue) and forest (green) habitat types for each study system (ANT = Antwerp, BUD = Budapest, GLA = Glasgow, GOT = Göteborg, HAR = Harjavalta, HEL = Helsingborg, Malmo = Malmö, MON = Montpellier, MUN = Munich, PAR = Paris, STR = Strasbourg, VES = Veszprém, WAR = Warsaw). Mean and range proportion ISA for each habitat type is shown above each plot.


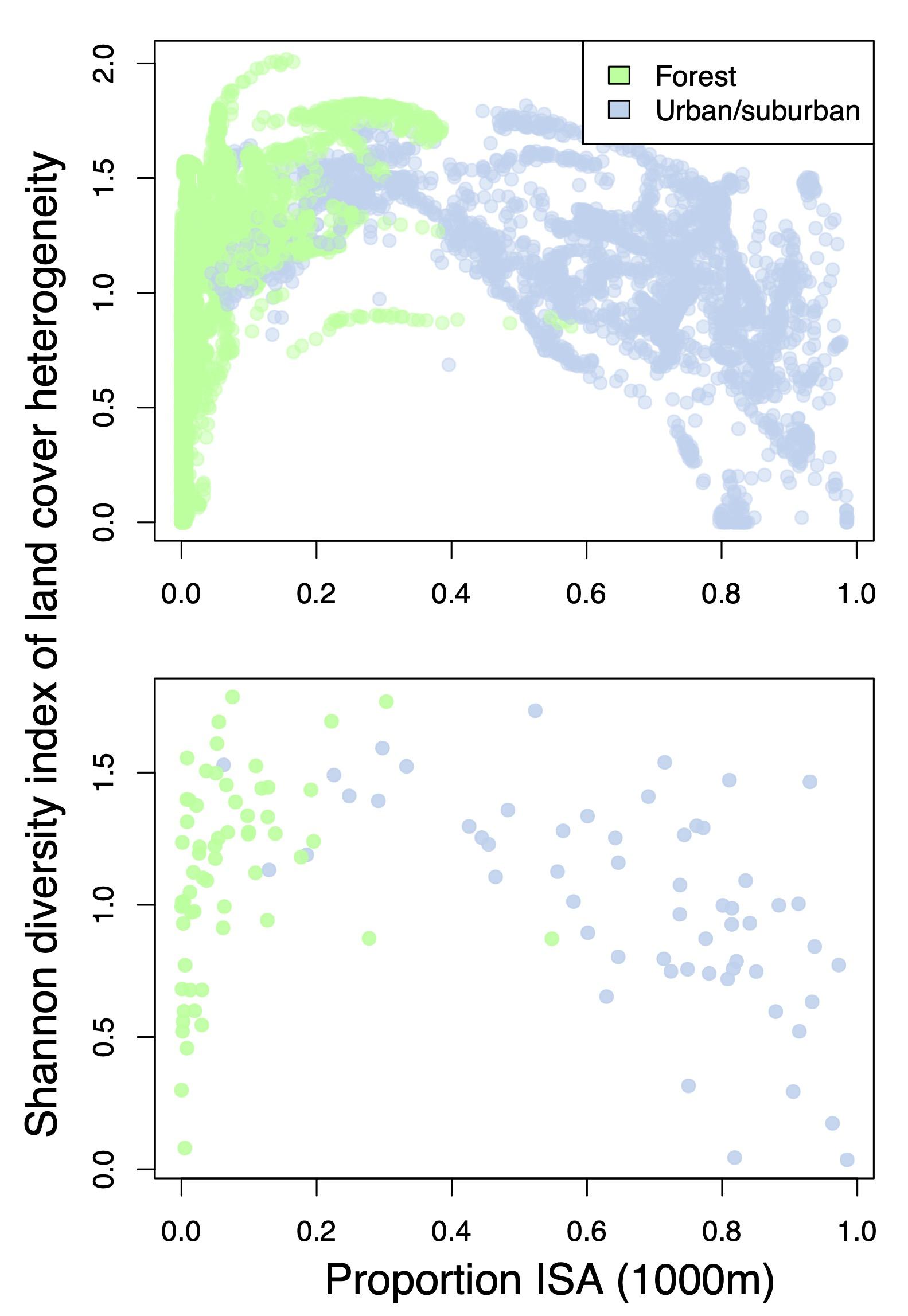


**Figure S5.** Relationship between the Shannon diversity index of land cover heterogeneity and the proportion of impervious surface area (ISA or urbanization) both at 1000m for top panel: each nest box (N = 7210) in the combined dataset and bottom panel: the average for each cluster (N = 119) in the dataset.

**Figure S6.** Mean habitat differences (forest = green, urban = blue) across European populations of great tits (squares) and blue tits (triangles) for A) adult tarsus length, B) nestling tarsus length, and C) lay date. Model estimates related to each parameter are back-transformed from Table 1 (back-transformed estimates presented in Table S7) and their 95% (thin line) highest posterior density intervals are shown. See Figure S7 for changes in mean traits along urbanization gradient of proportion ISA.


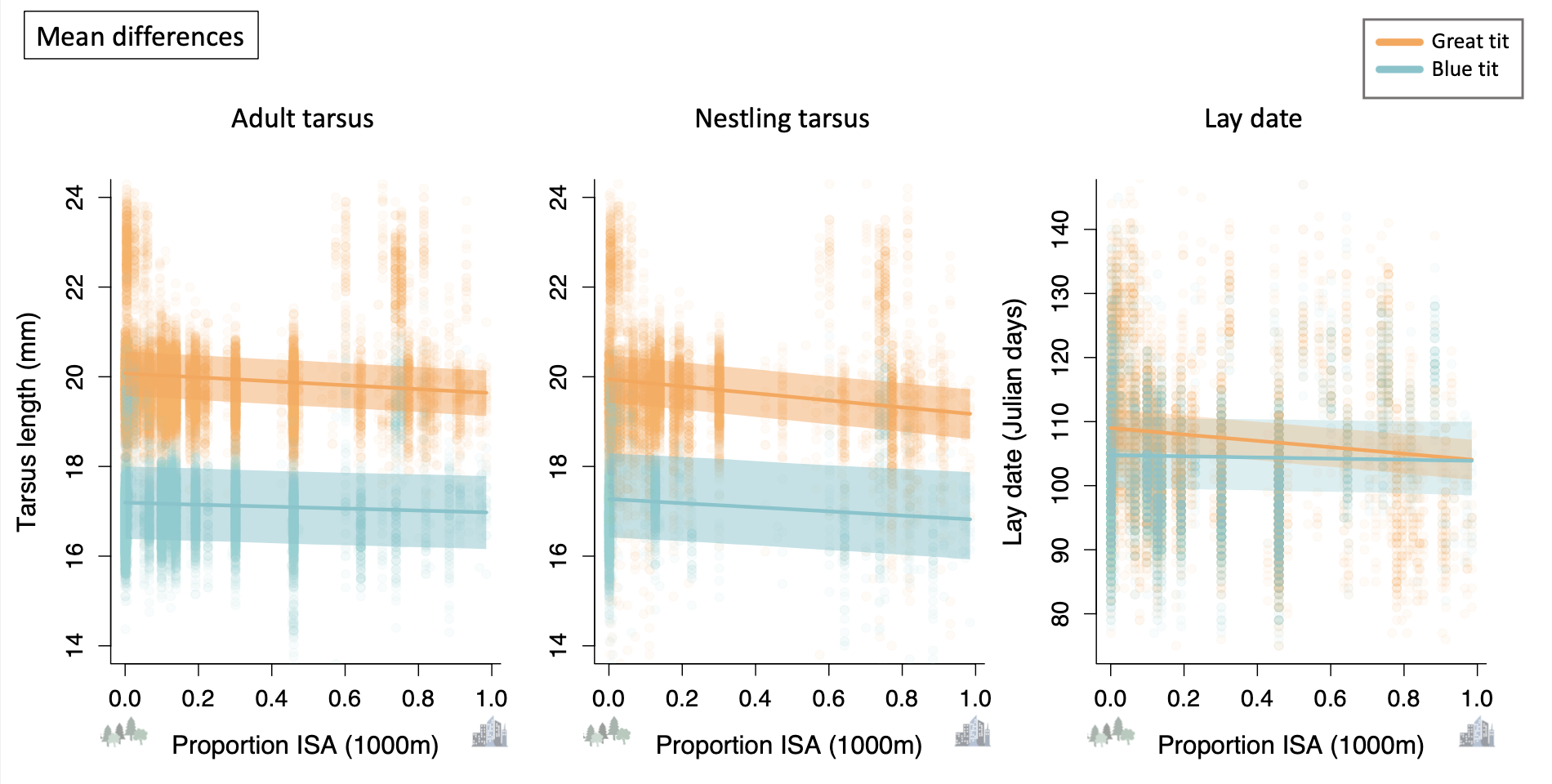


**Figure S7.** The effect of proportion of impervious surface area (ISA; higher values indicate higher urbanization) at 1000 meters on the mean adult tarsus length (left panel), mean nestling tarsus length (middle panel), and mean lay date (right panel; in Julian days since Jan 1) in great and blue tits (orange and blue, respectively). All traits showed statistically clear declines with ISA (credible intervals exclude zero; Table S5), except for lay date in blue tits where there was weaker evidence for an effect.


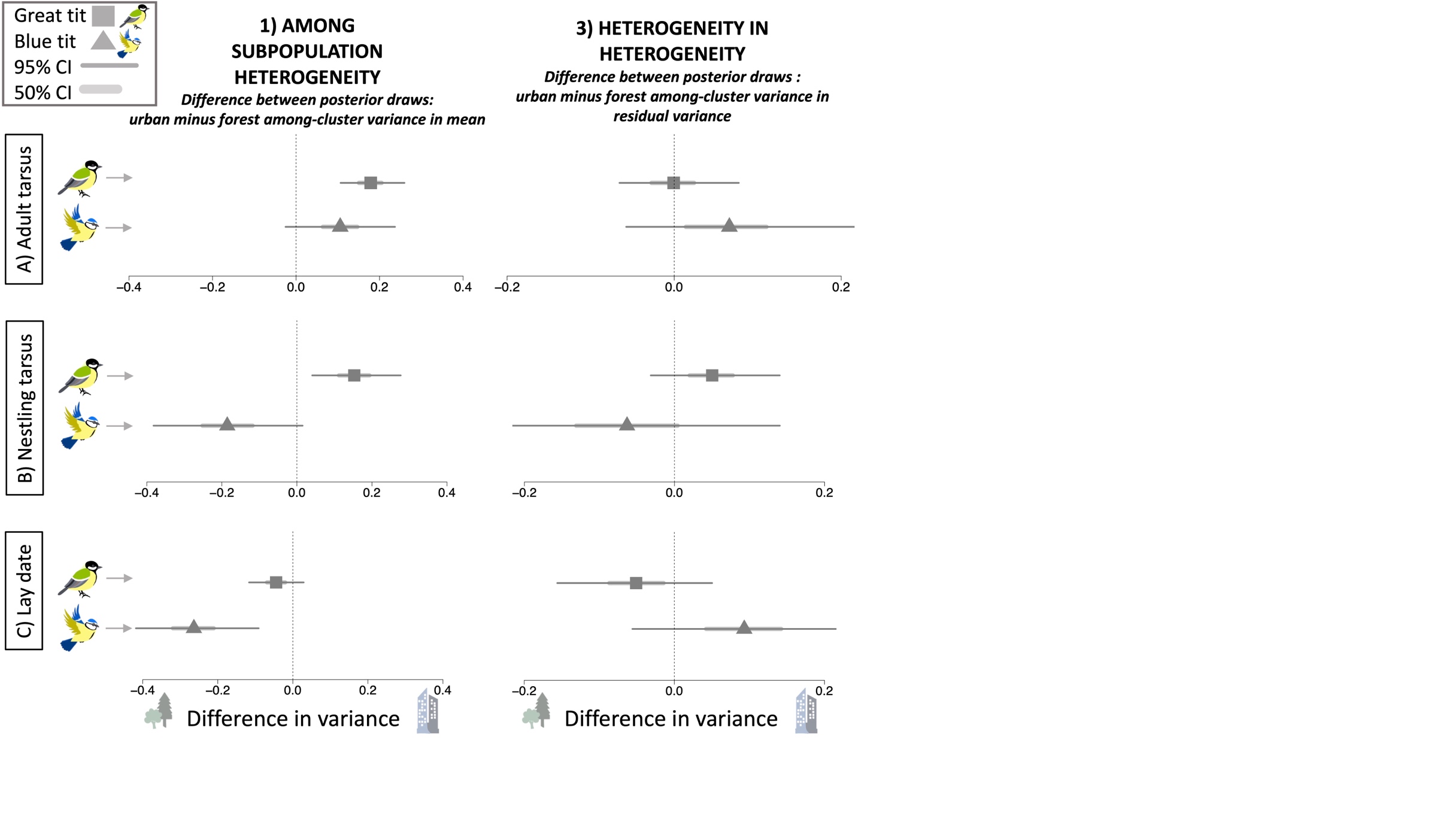


**Figure S8.** Results of the 1) Among-subpopulation heterogeneity hypothesis and 3) Heterogeneity in heterogeneity hypothesis for A) Adult tarsus, B) Nestling tarsus, and c) Lay date when calculating the difference between urban and forest among-cluster variance shown in Figure 3 in the main text (i.e., subtracting urban - forest posterior draws). Positive values indicate more variance among urban clusters and negative variance indicate more variance among forest clusters, while effects whose 95% (thin line) highest posterior density intervals exclude zero show evidence for a difference between urban and forest among-cluster variance. See also Figure 3 in main text which shows variance estimates separately for forest and urban habitat types as directly computed by the model.

**Section 4: Supplementary Tables**

**Table S1.** Summary of datasets used to examine patterns of phenotypic variation along urban gradients comprising urban (U) and forest (F) habitats across Europe in two species (GT = great tits, BT = blue tits) and across three traits (adult tarsus length = Tarsus, nestling tarsus length = Nestling tarsus, and lay date).

| Study system | Country | IMD2018 tile | # individuals  Tarsus, Nestling tarsus, Lay date  (GT = great tit, BT = blue tit) | Years (U/F)^1^ | (#) Urban sites / clusters^2^ | (#) Forest sites / clusters^3^ | # Nest boxes (U/F) | Tarsus method |
| --- | --- | --- | --- | --- | --- | --- | --- | --- |
| Harjavalta | Finland | E49N42_03035_v010  E49N43_03035_v010 | GT = NA, NA, 481  BT = NA, NA, 104 | 1991 – 2018/  1991 – 2018 | (12) 01, 02, 03, 04, 05, 17, 18, 19, 20, 21, 22, 25 / (8) 1:5, 17:19 | (12) 06, 07, 08, 09, 10, 11, 12, 13, 14, 15, 16, 23 / (12) 6:16, 20 | 181 / 257 | Alternative |
| Göteborg | Sweden | E44N38_03035_v010 | GT = 83, 25, 39  BT = 7, 4, 3 | 2017 – 2019/  2017 - 2019 | (2) RY, SL / (2) 81, 84 | (2) GR, VO / (2) 73, 89, | 46 / 41 | Alternative |
| Helsingborg | Sweden | E44N36_03035_v010 | GT = 148,84, 71  BT = 78, 43, 36 | 2018 – 2020/  2018 - 2020 | (2) AK, JB / (2) 71, 74, | (3) KB, SM, SB / (4) 76:77, 82, 85 | 66 / 51 | Alternative |
| Glasgow | Scotland | E34N37_03035_v010 | GT = 62, 107, 32  BT = 161, 276, 149 | 2014 – 2020/  2017 - 2020 | (2) GAR, KEL / (2) 58:59 | (3) CAS, SAL, SCE / (3) 55:57 | 87 / 186 | Alternative |
| Malmö | Sweden | E45N36_03035_v010 | GT = 897, 622, 4856  BT = 377, 282, 194 | 2013 – 2020/  2013 - 2020 | (5) S, K, P, R, RS / (4) 75,78:80 | (5) SK, DN, SW, TO, V / (5) 72,83,86,88 | 232 / 340 | Alternative |
| Warsaw | Poland | E50N32_03035_v010  E50N33_03035_v010 | GT = 450, 250, 196  BT = 524, 197, 259 | 2016 – 2020/  2016 - 2020 | (7) BIB, CMZ, LOL, MUR, OLO, POL, UNI / (5) 90:91, 93, 95:96 | (2) KPN, PAL / (2) 92, 94 | 262 / 103 | Alternative |
| Antwerp | Belgium | E39N31_03035_v010 | GT = 5934, 1946, 2713  BT = 4396, 70, 2212 | 1999 - 2022/  1994 - 2018 | (1) UA / (1) 50 | (2) BOS, PEE / (8) 42:49 | 216 / 636 | Alternative & Standard |
| Strasbourg | France | E41N28_03035_v010 | GT = 303, 332, 138 | 2014 – 2022/  2014 – 2022 | (11) ALPE, CAMP, CITA, CNRS, ESPL, HEYR, JARD, OBSE, QUIN, ROBES, ROUG / (8) 111:118 | (1) WANT / (1) 119 | 88 / 66 | Alternative |
| Paris | France | E37N28_03035_v010 | GT = 871, 621, 428  BT = 833, 468, 431 | 2012 – 2021/  2010 - 2021 | (10) PM, BU, CA, C,V, BC, BL, J, M, K / (9) 102:110 | (3) FOL, FON, COM / (5) 97:101 | 82 / 252 | Oxford |
| Munich | Germany | E44N27_03035_v010 | GT = 2503, 1950, 1094 | 2014 – 2015/  2010 - 2019 | (1) MUC / (1) 200^4^ | (12) 10, 11, 12, 13, 14, 15, 16, 17, 18, 19, 20, 21 / (12) 30:41 | 54 / 549 | Alternative |
| Budapest | Hungary | E49N27_03035_v010  E50N27_03035_v010 | GT = 133, 107, 65 | 2019 – 2021/  2020 - 2021 | (3) GES, VAR, VER / (3) 60:61, 65 | (1) ORD / (3) 62:64 | 49 / 32 | Alternative |
| Veszprém | Hungary | E49N26_03035_v010 | GT = 445, 529, 207 | 2013 – 2020/  2013 - 2020 | (1) VES / (3) 66:67, 70 | (2) VIL, GUL / (2) 68:69 | 84 / 88 | Alternative |
| Montpellier | France | E38N22_03035_v010  E38N23_03035_v010 | GT = 1080, 932, 458  BT = 2038, 1476, 1031 | 2007 – 2018/  1991 - 2018 | (8) BOT, CEF, FAC, FON, GRA, MAS, MOS, ZOO / (8) 21:27, 29 | (1) ROU / (1) 28 | 144 / 281 | Alternative |
| Barcelona | Spain | E36N20_03035_v010 | GT = 645, NA, NA  BT = NA, NA, NA | 1994 – 2018/  1998 – 2021 | (5) LAB, SET, SAR, ZOO, CIT / (3) 51:53 | (1) CAC / (1) 54 | 28 / 178 | Alternative |

^1^ Urban (U) and forest (F) year overlap are not the same across systems so this is max range,^2^Number of urban sites defined by data owners vs. number of urban clusters identified by clustering algorithm ^3^Same as ^2^ but for forest sites and clusters, ^4^Munich urban nest boxes did not cluster together as points in city are too far apart, so this cluster is defined manually and we test whether results change when excluding.**Table S2.** Summary of cluster characteristics across each study system for urban and forest clusters. ISA represents the proportion of impervious surface area (range = 0 – 1, where 1 = all ISA) and heterogeneity is the Shannon diversity index of the number of land cover types^1^ (range = 0 – 2).

| Study System | Habitat | # clusters | Mean ISA  (1000m) | Range ISA  (1000m) | Mean ISA  (100m) | Range ISA  (100m) | Mean Heterogeneity | Range heterogeneity | Mean area (m^2^) | Mean years of data collection |
| --- | --- | --- | --- | --- | --- | --- | --- | --- | --- | --- |
| Harjavalta | urban | 8 | 0.40 | 0.04 - 0.76 | 0.35 | 0 - 1 | 1.44 | 1.05 - 1.82 | 455737.27 | 24.32 |
|  | forest | 12 | 0.04 | 0 - 0.26 | 0.01 | 0 - 0.37 | 1.00 | 0.16 - 1.56 | 178081.42 | 23.87 |
| Göteborg | urban | 2 | 0.64 | 0.55 - 0.77 | 0.18 | 0 - 0.65 | 1.26 | 0.89 - 1.7 | 95621.73 | 3.60 |
|  | forest | 2 | 0.05 | 0.03 - 0.07 | 0.01 | 0 - 0.16 | 1.60 | 1.41 - 1.8 | 148195.74 | 3.70 |
| Helsingborg | urban | 2 | 0.71 | 0.59 - 0.82 | 0.14 | 0 - 0.53 | 1.16 | 0.75 - 1.36 | 72709.29 | 3.51 |
|  | forest | 4 | 0.04 | 0 - 0.11 | 0.00 | 0 - 0.03 | 0.98 | 0.62 - 1.3 | 133695.72 | 3.22 |
| Glasgow | urban | 2 | 0.77 | 0.51 - 0.91 | 0.49 | 0.02 - 1 | 0.96 | 0.68 - 1.34 | 487630.29 | 7.00 |
|  | forest | 3 | 0.00 | 0 - 0.01 | 0.01 | 0 - 0.13 | 1.12 | 0.75 - 1.54 | 247665.30 | 8.82 |
| Malmö | urban | 4 | 0.76 | 0.68 - 0.94 | 0.26 | 0 - 0.89 | 1.09 | 0.17 - 1.5 | 359894.48 | 7.96 |
|  | forest | 5 | 0.01 | 0 - 0.11 | 0.00 | 0 - 0.09 | 0.84 | 0 - 1.39 | 1819914.87 | 3.57 |
| Warsaw | urban | 5 | 0.64 | 0.13 - 0.92 | 0.38 | 0 - 1 | 0.89 | 0 - 1.6 | 350636.23 | 4.44 |
|  | forest | 2 | 0.03 | 0 - 0.16 | 0.06 | 0 - 0.57 | 0.94 | 0.54 - 1.52 | 385092.60 | 4.65 |
| Antwerp | urban | 1 | 0.46 | 0.28 - 0.61 | 0.29 | 0 - 1 | 1.11 | 0.68 - 1.65 | 1595673.00 | 26.00 |
|  | forest | 8 | 0.16 | 0.05 - 0.39 | 0.02 | 0 - 0.32 | 1.44 | 1.17 - 1.82 | 141088.22 | 21.67 |
| Strasbourg | urban | 8 | 0.69 | 0.04 - 0.95 | 0.48 | 0 - 1 | 1.03 | 0.26 - 1.51 | 334457.37 | 6.93 |
|  | forest | 1 | 0.10 | 0.01 - 0.38 | 0.00 | 0 - 0.16 | 1.27 | 1.02 - 1.4 | 634267.30 | 9.00 |
| Paris | urban | 9 | 0.86 | 0.41 - 0.99 | 0.64 | 0.05 - 1 | 0.70 | 0 - 1.36 | 57649.88 | 9.47 |
|  | forest | 5 | 0.05 | 0 - 0.24 | 0.01 | 0 - 0.15 | 0.85 | 0.04 - 1.71 | 495775.74 | 11.22 |
| Munich | urban | 1 | 0.80 | 0.29 - 0.97 | 0.88 | 0.11 - 1 | 0.91 | 0.02 - 1.52 | 110806.06 | 2.00 |
|  | forest | 12 | 0.07 | 0 - 0.3 | 0.00 | 0 - 0.33 | 1.25 | 0.29 - 1.79 | 70248.00 | 9.81 |
| Budapest | urban | 3 | 0.80 | 0.71 - 0.86 | 0.44 | 0.07 - 0.94 | 0.87 | 0.68 - 1.06 | 34868.44 | 2.88 |
|  | forest | 3 | 0.29 | 0.1 - 0.58 | 0.06 | 0 - 0.23 | 0.88 | 0.74 - 0.99 | 419612.46 | 1.72 |
| Veszprém | urban | 3 | 0.75 | 0.55 - 0.87 | 0.59 | 0.25 - 0.98 | 0.83 | 0.54 - 1.33 | 221298.71 | 7.81 |
|  | forest | 2 | 0.02 | 0 - 0.17 | 0.00 | 0 - 0.03 | 1.24 | 0.7 - 2.02 | 384684.49 | 6.82 |
| Montpellier | urban | 8 | 0.53 | 0.13 - 0.94 | 0.49 | 0 - 1 | 1.08 | 0.2 - 1.75 | 2865311.15 | 7.74 |
|  | forest | 1 | 0.00 | 0 - 0 | 0.00 | 0 - 0 | 0.68 | 0 - 1.25 | 455737.27 | 28.00 |
| Barcelona | urban | 3 | 0.56 | 0.24 - 0.87 | 0.24 | 0 - 0.65 | 1.17 | 0.7 - 1.5 | 101870.3 | 25.02 |
|  | forest | 1 | 0.00 | 0 - 0.03 | 0.00 | 0 - 0 | 0.08 | 0 - 0.25 | 690549.5 | 24 |

^1^Of the 44 possible Corine land cover types, the spatial extent of the combined dataset included 29 land cover types including: continuous urban fabric, discontinuous urban fabric, industrial or commercial units, road and rail networks, port areas, mineral extraction sites, dump sites, construction sites, green urban areas, sport and leisure facilities, non-irrigated arable land, vineyards, pastures, complex cultivation patterns, agricultural areas with significant natural vegetation, agro-forestry areas, broad-leaved forest, coniferous forest, mixed forest, natural grasslands, moors and heathland, sclerophyllous vegetation, transitional woodland shrub, sparsely vegetated area, inland marshes, peat bog, water courses, water bodies, estuaries, and sea and ocean.

**Table S3.** Number of urban and forest clusters (i.e., subpopulations) for each study system defined using different clustering algorithm definitions compared to the number of study areas defined by data owners. Clusters were defined as groups of at least 5 nestboxes that were within a distance of at least 200, 300, or 400 meters. In most cases, clusters defined using 300m distances (bolded column) tended to be more comparable to owner-defined study areas and, when inspected visually, tended to be contained within similar types of habitats. Note that Munich urban nest boxes did not cluster together under any definition, so this urban cluster was added manually.

| Study System | Habitat | Number of study areas | Clusters (5+ nest boxes within X meters) | | |
| --- | --- | --- | --- | --- | --- |
|  |  |  | X = 200 m | **X = 300 m** | X = 400 m |
| Harjavalta | urban | 12 | 10 | 8 | 7 |
|  | forest | 12 | 13 | 12 | 12 |
| Göteborg | urban | 2 | 2 | 2 | 2 |
|  | forest | 2 | 2 | 2 | 2 |
| Helsingborg | urban | 2 | 2 | 2 | 2 |
|  | forest | 3 | 5 | 4 | 4 |
| Glasgow | urban | 2 | 2 | 2 | 2 |
|  | forest | 3 | 3 | 3 | 2 |
| Malmö | urban | 5 | 4 | 4 | 4 |
|  | forest | 5 | 5 | 4 | 3 |
| Warsaw | urban | 7 | 6 | 5 | 5 |
|  | forest | 2 | 3 | 2 | 2 |
| Antwerp | urban | 1 | 1 | 1 | 1 |
|  | forest | 2 | 9 | 8 | 4 |
| Strasbourg | urban | 11 | 14 | 8 | 5 |
|  | forest | 1 | 1 | 1 | 1 |
| Paris | urban | 10 | 9 | 9 | 9 |
|  | forest | 3 | 5 | 5 | 4 |
| Munich | urban | 1 | NA | NA | NA |
|  | forest | 12 | 12 | 12 | 12 |
| Budapest | urban | 3 | 3 | 3 | 3 |
|  | forest | 1 | 3 | 3 | 1 |
| Veszprém | urban | 1 | 4 | 3 | 3 |
|  | forest | 2 | 2 | 2 | 2 |
| Montpellier | urban | 8 | 11 | 8 | 8 |
|  | forest | 1 | 1 | 1 | 1 |
| Barcelona | urban | 5 | 3 | 3 | 3 |
|  | forest | 1 | 1 | 1 | 1 |
| **TOTAL** |  | **120** | **136** | **119** | **105** |

**Table S4.** Model estimates and their 95% highest posterior density intervals (HPDIs) for adult tarsus length when instead selecting a random observation per individual. Effects highlighted in bold have changed conclusions (i.e., HDPI now overlaps or not zero) from Table 1 in main text.

|  | A) Adult tarsus | |
| --- | --- | --- |
| **Mean part** | Great tit  N = 13557 | Blue tit  N = 8411 |
| *Fixed effects* |  |  |
| Intercept (*β*_m0_) | 0.213 [-0.289, 0.715] | 0.235 [-0.601, 1.121] |
| Habitat (urban) | -0.295 [-0.38, -0.212] | -0.188 [-0.28, -0.099] |
| Latitude | 0.61 [0.272, 0.93] | 0.521 [-0.057, 1.062] |
| Sex (male) | 0.536 [0.518, 0.554] | 0.589 [0.565, 0.614] |
| *Random Effects* |  |  |
| Year | 0.03 [0.016, 0.047] | 0.242 [0.187, 0.315] |
| System | 0.89 [0.585, 1.394] | 1.17 [0.703, 1.927] |
| Cluster: |  |  |
| Forest | 0.056 [0.033, 0.085] | 0.037 [0.002, 0.093] |
| Urban | 0.23 [0.166, 0.31] | 0.138 [0.027, 0.257] |
| **Dispersion part** |  |  |
| *Fixed effects* |  |  |
| Intercept (*βv*_0,exp_) | -0.535 [-0.682, -0.384] | -0.548 [-0.862, -0.181] |
| Habitat (urban) | 0.04 [-0.025, 0.102] | 0.129 [0.048, 0.207] |
| Heterogeneity (1000m) | -0.004 [-0.035, 0.028] | 0.003 [-0.036, 0.044] |
| Latitude | 0.042 [-0.057, 0.145] | 0.275 [0.037, 0.487] |
| Cluster area | 0.053 [0.034, 0.072] | 0.135 [0.023, 0.23] |
| Cluster years | 0.008 [-0.054, 0.07] | 0.006 [-0.09, 0.101] |
| Sex (male) | 0.02 [-0.004, 0.045] | -0.002 [-0.034, 0.029] |
| *Random effects* |  |  |
| System (intercept) | 0.231 [0.139, 0.392] | 0.347 [0.136, 0.765] |
| System (*r*_mean, dispersion_) | -0.074 [-0.557, 0.45] | -0.139 [-0.689, 0.487] |
| Cluster: |  |  |
| Forest (intercept) | 0.088 [0.056, 0.129] | 0.035 [0.001, 0.097] |
| Forest (*r*_mean, dispersion_) | -0.377 [-0.799, 0.176] | -0.086 [-0.835, 0.769] |
| Urban (intercept) | 0.07 [0.008, 0.134] | 0.072 [0.003, 0.191] |
| Urban (*r*_mean, dispersion_) | -0.208 [-0.775, 0.442] | -0.129 [-0.834, 0.709] |

**Table S5.** Model estimates and their 95% highest posterior density intervals (HPDIs) when examining the effect of continuous urbanization (i.e., impervious surface area; ISA) at 1000 meters instead of the categorical effect of habitat (i.e., urban vs. forest). Effects highlighted in bold differ from Table 1 in main text in their statistical support (i.e., whether HPDI overlaps or not zero) for A) adult tarsus length and B) nestling tarsus length, and C) female lay dates.

|  | A) Adult tarsus | | B) Nestling tarsus | | C) Lay date | |
| --- | --- | --- | --- | --- | --- | --- |
| **Mean model** | Great tit | Blue tit | Great tit | Blue tit | Great tit | Blue tit |
| *Fixed effects* |  |  |  |  |  |  |
| Intercept (*β*_m0_) | 0.132 [-0.369, 0.604] | 0.162 [-0.696, 1.019] | -1.146 [-1.674, -0.598] | -0.693 [-1.702, 0.345] | 0.177 [-0.076, 0.431] | 0.242 [-0.222, 0.746] |
| ISA 1000 m | -0.108 [-0.13, -0.087] | -0.065 [-0.091, -0.04] | -0.166 [-0.205, -0.127] | **-0.086 [-0.158, -0.015]** | -0.102 [-0.131, -0.073] | -0.021 [-0.072, 0.029] |
| Latitude | 0.673 [0.356, 0.988] | 0.551 [-0.038, 1.09] | 0.554 [0.215, 0.866] | 0.572 [-0.227, 1.291] | 0.665 [0.485, 0.841] | 0.718 [0.38, 0.999] |
| A) Sex (male), B) Chick age | 0.545 [0.527, 0.563] | 0.594 [0.569, 0.618] | 0.085 [0.065, 0.104] | 0.048 [0.016, 0.08] |  |  |
| *Random Effects* |  |  |  |  |  |  |
| Breeding Season | 0.038 [0.024, 0.055] | 0.239 [0.184, 0.315] | 0.111 [0.08, 0.154] | 0.132 [0.091, 0.186] | 0.396 [0.307, 0.516] | 0.507 [0.395, 0.668] |
| System | 0.908 [0.607, 1.379] | 1.211 [0.74, 1.949] | 0.753 [0.48, 1.192] | 1.053 [0.62, 1.775] | 0.37 [0.24, 0.582] | 0.536 [0.298, 0.996] |
| Clusters | 0.098 [0.07, 0.132] | 0.072 [0.042, 0.112] | 0.17 [0.127, 0.221] | 0.254 [0.172, 0.349] | 0.136 [0.109, 0.167] | 0.225 [0.165, 0.294] |
| **Dispersion model** |  |  |  |  |  |  |
| *Fixed effects* |  |  |  |  |  |  |
| Intercept (*βv*_0,exp_) | -0.539 [-0.684, -0.383] | -0.526 [-0.866, -0.14] | -0.4 [-0.581, -0.204] | -0.44 [-0.655, -0.231] | -0.708 [-0.813, -0.596] | -0.485 [-0.738, -0.267] |
| ISA 1000 m | 0.013 [-0.007, 0.033] | 0.025 [-0.005, 0.055] | 0.059 [0.022, 0.096] | 0.084 [0.022, 0.151] | 0.043 [0.008, 0.076] | 0.013 [-0.03, 0.054] |
| Heterogeneity 1000 m | -0.012 [-0.037, 0.013] | 0.011 [-0.035, 0.06] | <0.001 [-0.038, 0.04] | 0.005 [-0.066, 0.08] | 0.024 [-0.016, 0.063] | -0.004 [-0.065, 0.055] |
| Latitude | 0.046 [-0.051, 0.151] | 0.29 [0.04, 0.55] | 0.091 [-0.042, 0.23] | 0.076 [-0.093, 0.223] | -0.032 [-0.105, 0.043] | -0.024 [-0.17, 0.122] |
| Cluster area | 0.032 [0.016, 0.049] | 0.132 [0.002, 0.253] | 0.008 [-0.023, 0.04] | 0.222 [-0.007, 0.458] | -0.014 [-0.073, 0.045] | -0.011 [-0.163, 0.127] |
| Cluster years | -0.006 [-0.054, 0.043] | -0.006 [-0.118, 0.108] | 0.013 [-0.065, 0.085] | -0.163 [-0.348, 0.012] | 0.021 [-0.043, 0.088] | 0.079 [-0.041, 0.18] |
| A) Sex (male), C) Age (1) | 0.021 [-0.003, 0.045] | -0.005 [-0.037, 0.025] |  |  | -0.032 [-0.075, 0.009] | -0.035 [-0.088, 0.016] |
| *Random effects* |  |  |  |  |  |  |
| System (intercept) | 0.244 [0.149, 0.399] | 0.372 [0.152, 0.782] | 0.281 [0.159, 0.494] | 0.098 [0.004, 0.338] | 0.13 [0.059, 0.235] | 0.208 [0.065, 0.469] |
| System (*r*_mean,dispersion_) | -0.105 [-0.592, 0.402] | -0.136 [-0.689, 0.495] | -0.141 [-0.65, 0.419] | -0.057 [-0.776, 0.718] | 0.383 [-0.22, 0.818] | 0.327 [-0.369, 0.858] |
| Clusters (intercept) | 0.067 [0.04, 0.097] | 0.073 [0.034, 0.123] | 0.136 [0.099, 0.178] | 0.178 [0.09, 0.278] | 0.131 [0.092, 0.174] | 0.114 [0.031, 0.204] |
| Clusters (*r*_mean,dispersion_) | -0.576 [-0.882, -0.12] | -0.352 [-0.854, 0.328] | 0.679 [-0.887, -0.378] | -0.542 [-0.896, -0.022] | 0.004 [-0.34, 0.348] | 0.208 [-0.353, 0.646] |

**Table S6.** Model estimates and their 95% highest posterior density intervals (HPDIs) when examining the effect of continuous urbanization (i.e., impervious surface area; ISA) at 100 meters instead of the categorical effect of habitat (i.e., urban vs. forest). Effects highlighted in bold differ from Table 1 in main text in their statistical clarity (i.e., whether HPDI overlaps or not zero) for A) adult tarsus length and B) nestling tarsus length, and C) female lay dates from Table 1 in main text.

|  | A) Adult tarsus | | B) Nestling tarsus | | C) Lay date | |
| --- | --- | --- | --- | --- | --- | --- |
| **Mean model** | Great tit | Blue tit | Great tit | Blue tit | Great tit | Blue tit |
| *Fixed effects* |  |  |  |  |  |  |
| Intercept (*β*_m0_) | 0.102 [-0.41, 0.593] | 0.13 [-0.718, 0.974] | -1.171 [-1.701, -0.624] | -0.733 [-1.688, 0.275] | 0.175 [-0.079, 0.442] | 0.256 [-0.215, 0.768] |
| ISA 100 m | -0.086 [-0.114, -0.059] | -0.058 [-0.08, -0.036] | -0.136 [-0.185, -0.091] | **-0.071 [-0.131, -0.014]** | -0.098 [-0.126, -0.071] | -0.037 [-0.082, 0.008] |
| Latitude | 0.641 [0.31, 0.974] | 0.59 [0.038, 1.123] | 0.552 [0.229, 0.876] | 0.577 [-0.149, 1.243] | 0.655 [0.465, 0.828] | 0.718 [0.393, 1] |
| A) Sex (male), B) Chick age | 0.545 [0.527, 0.563] | 0.594 [0.57, 0.618] | 0.086 [0.066, 0.105] | 0.049 [0.017, 0.081] |  |  |
| *Random Effects* |  |  |  |  |  |  |
| Breeding Season | 0.038 [0.024, 0.056] | 0.239 [0.183, 0.314] | 0.112 [0.081, 0.154] | 0.132 [0.09, 0.184] | 0.395 [0.308, 0.513] | 0.505 [0.391, 0.652] |
| System | 0.897 [0.593, 1.361] | 1.176 [0.725, 1.936] | 0.729 [0.461, 1.171] | 1.029 [0.604, 1.758] | 0.385 [0.246, 0.626] | 0.527 [0.297, 0.947] |
| Clusters | 0.146 [0.115, 0.183] | 0.063 [0.034, 0.103] | 0.216 [0.167, 0.274] | 0.251 [0.169, 0.348] | 0.134 [0.108, 0.165] | 0.223 [0.164, 0.299] |
| **Dispersion model** |  |  |  |  |  |  |
| *Fixed effects* |  |  |  |  |  |  |
| Intercept (*βv*_0,exp_) | -0.535 [-0.675, -0.376] | -0.511 [-0.863, -0.118] | -0.403 [-0.582, -0.214] | -0.445 [-0.644, -0.24] | -0.719 [-0.823, -0.605] | -0.48 [-0.698, -0.27] |
| ISA 100 m | 0.011 [-0.009, 0.032] | **0.019 [-0.006, 0.045]** | 0.051 [0.012, 0.09] | 0.07 [0.024, 0.121] | 0.035 [0.004, 0.067] | 0.022 [-0.013, 0.057] |
| Heterogeneity 100m | -0.008 [-0.029, 0.013] | -0.018 [-0.051, 0.016] | 0.009 [-0.027, 0.045] | -0.004 [-0.059, 0.048] | 0.006 [-0.031, 0.044] | -0.032 [-0.081, 0.014] |
| Latitude | 0.044 [-0.057, 0.151] | 0.29 [0.043, 0.545] | 0.088 [-0.037, 0.226] | 0.107 [-0.057, 0.252] | -0.027 [-0.103, 0.048] | -0.022 [-0.156, 0.103] |
| Cluster area | 0.032 [0.016, 0.05] | **0.103 [-0.026, 0.213]** | 0.004 [-0.032, 0.037] | 0.211 [-0.002, 0.421] | -0.025 [-0.08, 0.031] | -0.018 [-0.153, 0.117] |
| Cluster years | -0.002 [-0.053, 0.045] | 0.012 [-0.093, 0.123] | 0.007 [-0.073, 0.084] | -0.141 [-0.326, 0.038] | 0.015 [-0.047, 0.081] | 0.094 [-0.012, 0.187] |
| A) Sex (male), C) Age (1) | 0.021 [-0.003, 0.044] | -0.005 [-0.036, 0.026] |  |  | -0.032 [-0.074, 0.01] | -0.034 [-0.086, 0.016] |
| *Random effects* |  |  |  |  |  |  |
| System (intercept) | 0.246 [0.151, 0.413] | 0.388 [0.162, 0.821] | 0.267 [0.152, 0.467] | 0.089 [0.003, 0.315] | 0.133 [0.059, 0.243] | 0.18 [0.048, 0.432] |
| System (*r*_mean,dispersion_) | -0.108 [-0.584, 0.415] | -0.126 [-0.677, 0.508] | -0.119 [-0.618, 0.417] | 0.007 [-0.757, 0.753] | 0.336 [-0.247, 0.778] | 0.376 [-0.376, 0.894] |
| Clusters (intercept) | 0.065 [0.04, 0.095] | 0.072 [0.032, 0.123] | 0.141 [0.102, 0.186] | 0.169 [0.088, 0.27] | 0.129 [0.09, 0.172] | 0.11 [0.034, 0.191] |
| Clusters (*r*_mean,dispersion_) | -0.49 [-0.802, -0.046] | -0.472 [-0.918, 0.215] | -0.643 [-0.846, -0.358] | -0.528 [-0.886, -0.011] | 0.041 [-0.31, 0.376] | 0.139 [-0.423, 0.601] |

**Table S7.** Back-transformed fixed-effect estimates and their 95% highest posterior density intervals (HPDIs) from Table 1 in the main text. Fixed and random effect estimates specified in the mean and dispersion (i.e., explains residual variation) parts of a double hierarchical linear model (DHGLM) when examining the effect of urbanization (forest vs. urban) on the mean and residual variation of three traits: A) adult tarsus length, B) nestling tarsus length (in mm), and C) female lay dates (in days). Great and blue tit data were run in separate models (*N* = number of individuals / observations shown for each). Since dispersion estimates are estimated on the log scale, linear estimates of continuous variables (i.e., heterogeneity, latitude, cluster area and years) are no longer linear when transforming to the data scale, so these effects are not shown.

|  | A) Adult tarsus | | B) Nestling tarsus | | C) Lay date | |
| --- | --- | --- | --- | --- | --- | --- |
| **Mean part** | Great tit  N = 13554 | Blue tit  N = 8414 | Great tit  N = 7505 | Blue tit  N = 2905 | Great tit  N = 6378 | Blue tit  N = 4419 |
| *Fixed effects* |  |  |  |  |  |  |
| Intercept (*β*_m0_) | 20.13 [19.61, 20.64] | 17.21 [16.45, 17.94] | 18.31 [17.61, 19.03] | 16.26 [15.2, 17.39] | 108.59 [105.45, 111.86] | 104.5 [99.14, 110.17] |
| Habitat (urban) | 19.83 [19.21, 20.41] | 17.05 [16.21, 17.86] | 17.79 [16.93, 18.67] | 16.09 [14.81, 17.42] | 105.58 [101.53, 109.8] | 103.36 [96.56, 110.46] |
| Latitude | 0.19 [0.09, 0.28] | 0.12 [-0.01, 0.25] | 0.2 [0.06, 0.32] | 0.13 [-0.04, 0.27] | 1.94 [1.4, 2.43] | 1.8 [0.86, 2.61] |
| A) Sex (male), B) Chick age | 20.69 [20.15, 21.22] | 17.72 [16.94, 18.47] | 0.14 [0.1, 0.17] | 0.06 [0.02, 0.09] |  |  |
| **Dispersion part** |  |  |  |  |  |  |
| *Fixed effects* |  |  |  |  |  |  |
| Intercept (*βv*_0,exp_) | 0.6 [0.51, 0.7] | 0.49 [0.36, 0.7] | 0.83 [0.68, 1] | 0.65 [0.51, 0.81] | 5.64 [5.05, 6.39] | 6.06 [4.92, 7.45] |
| Habitat (urban; parameter 2) | 0.62 [0.51, 0.77] | 0.55 [0.36, 0.86] | 0.92 [0.69, 1.24] | 0.81 [0.54, 1.21] | 6.14 [5.05 – 7.61] | 6.65 [4.77, 9.22] |
| A) Sex (male), C) Age (1) | 0.61 [0.51, 0.73] | 0.49 [0.35, 0.73] |  |  | 5.47 [4.69 – 6.47] | 5.85 [4.5, 7.6] |

**Table S8.** Model estimates and their 95% highest posterior density intervals (HPDIs) when not including the city of Munich cluster. Effects highlighted in bold differ from Table 1 in main text in their statistical clarity (i.e., whether HPDI overlaps or not zero) for A) adult tarsus length and B) nestling tarsus length from Table 1 in main text.

|  | 1. Adult tarsus | 1. Nestling tarsus |
| --- | --- | --- |
| **Mean model** | Great tit | Great tit |
| *Fixed effects* |  |  |
| Intercept (*β*_m0_) | 0.204 [-0.307, 0.709] | -2.215 [-2.738, -1.706] |
| Habitat (urban) | -0.299 [-0.381, -0.212] | -0.618 [-0.771, -0.467] |
| Latitude | 0.616 [0.284, 0.943] | **0.179 [-0.153, 0.492]** |
| A) Sex (male), B) Chick age | 0.545 [0.527, 0.562] | 0.169 [0.143, 0.195] |
| *Random Effects* |  |  |
| Breeding Season | 0.038 [0.024, 0.056] | 0.154 [0.108, 0.219] |
| System | 0.903 [0.604, 1.396] | 0.46 [0.238, 0.888] |
| Clusters: |  |  |
| Forest | 0.053 [0.032, 0.081] | 0.153 [0.091, 0.237] |
| Urban | 0.235 [0.167, 0.318] | 0.206 [0.09, 0.344] |
| **Dispersion model** |  |  |
| *Fixed effects* |  |  |
| Intercept (*βv*_0,exp_) | -0.544 [-0.699, -0.39] | -0.113 [-0.421, 0.179] |
| Habitat (urban) | 0.047 [-0.011, 0.101] | 0.19 [0.054, 0.331] |
| Heterogeneity 1000m | -0.006 [-0.034, 0.021] | 0.001 [-0.039, 0.042] |
| Latitude | 0.043 [-0.054, 0.147] | 0.105 [-0.116, 0.336] |
| Cluster area | **0.282 [-0.405, 0.957]** | 0.511 [-1.319, 2.282] |
| Cluster years | -0.012 [-0.067, 0.044] | 0.01 [-0.09, 0.109] |
| A) Sex (male), C) Age (1) | 0.022 [-0.002, 0.046] |  |
| *Random effects* |  |  |
| System (intercept) | 0.241 [0.144, 0.403] | 0.302 [0.152, 0.605] |
| System (*r*_mean,dispersion_) | -0.11 [-0.59, 0.428] | -0.281 [-0.8, 0.396] |
| Clusters: |  |  |
| Forest (intercept) | 0.062 [0.031, 0.101] | 0.115 [0.072, 0.174] |
| Forest (*r*_mean,dispersion_) | -0.417 [-0.853, 0.182] | -0.687 [-0.946, -0.235] |
| Urban (intercept) | 0.058 [0.005, 0.126] | 0.152 [0.069, 0.259] |
| Urban (*r*_mean,dispersion_) | -0.301 [-0.858, 0.48] | -0.605 [-0.957, 0.015] |

**Table S9.** Summary and descriptions of model terms for supplementary model equations (presented in section 5) used to evaluate changes in mean and variation of adult tarsus length in response to urbanization using double hierarchical linear mixed models where fixed and random effects are fit in the mean and dispersion (i.e., to explain residual variance) parts of the model. Model equations for nestling tarsus and lay date are not shown since model structures are very similar.

| Model notation | | Random effects | | Fixed effects (*X_ij_*) | | Type^1^ |
| --- | --- | --- | --- | --- | --- | --- |
| *y_ijkln_* | Phenotypic value of trait *y* for the *l*th system, the *n*th year, the *k*th cluster, and the *j*th habitat at individual (instance) *i* | CLU | clusters (k) | HABITAT | Habitat type (j) of cluster *k* where X_k_ = 0 for forest and 1 for urban | CAT |
| *β_m_* | Estimate from mean model where *β_m0_* is population intercept | SYST | Study system (l) | LAT | Latitude of cluster *k* where X_k_ = 0 is mean latitude | CONT |
| *β_v,exp_* | Estimate (on ln scale) from dispersion model where *βv_0,exp_* is population intercept | YEAR | Year of each breeding season (n) | SEX | Sex of system *l,* for year *n,* at cluster *k,* of individual *i* where X_ikln_ = 0 for females and 1 for males | CAT |
| *e_ikln_* | Residual error or difference between predicted and fitted value for the *l*th system, the *n*th year, and the *k*th cluster at individual (instance) *i* |  |  | HET | Land cover heterogeneity of cluster *k* where X_k_ = 0 is mean heterogeneity | CONT |
| ρ(a,b) | Correlation between two random effect variables |  |  | AREA | Area (m^2^) of cluster *k* where X_k_ = 0 is mean area | CONT |
|  |  |  |  | YEARS | Number of years of data collection of cluster *k* where X_k_ = 0 is mean number of years | CONT |

^1^Type of variable where CAT = categorical, CONT = continuous

**Section 5: Supplementary model equations for adult tarsus length**

Following the format presented in O’Dea et al. (2022) and model terms outlined in Table S9, we present mathematical model equations of the mean part of the model (Eq.S3), the dispersion part of the model (Eq.S4), and their covariance for the analysis of adult tarsus length (Eq.S7-8). Parameter 1 is shown in blue (among-cluster variance in mean trait), parameter 2 is shown in red (slope coefficient of urbanization on within-cluster residual variance), and parameter 3 is shown in green (among-cluster variance in within-cluster residual variance).

| $y_{ijkln} =(\beta_{m0}+ {CLU}_{m0kj}+ {SYST}_{m0l} {+ YEAR}_{m0n}) + \beta_{m1}{HABITAT}_{k} + \beta_{m2}{LAT}_{k} + \beta_{m3}{SEX}_{ikln} + e_{ikln}$ | [Eq.S3] |
| --- | --- |
| $ln\left( \sigma_{e_{ikln}}^{2} \right)=(\beta_{v0,exp}+{CLU}_{v0kj,exp}+ {SYST}_{v0l,exp} {+ YEAR}_{v0n,exp})+ \beta_{v1,exp}{HABITAT}_{k} + \beta_{v2,exp}{HET}_{k} + \beta_{v3,exp}{LAT}_{k} +$  $\beta_{v4,exp}{AREA}_{k} + \beta_{v5,exp}{YEARS}_{k} + \beta_{v6,exp}{SEX}_{kln}$ | [Eq.S4] |
| $e_{ikln} \sim N(0,\sigma_{e_{ikln}}^{2})$ | [Eq.S5] |
| ${YEAR}_{n} \sim N(0, \sigma_{{YEAR}_{n}}^{2})$ | [Eq.S6] |
| ${SYST}_{l} \sim N_{2}\left( 0, \begin{matrix} {\sigma^{2}}_{{SYST}_{m0}} & \rho\left( {SYST}_{m0j},{SYST}_{v0j,exp} \right)\sigma_{{SYST}_{m0j}}\sigma_{{SYST}_{v0j,exp}} \\ \cdots& {\sigma^{2}}_{{SYST}_{v0,exp}} \end{matrix} \right)$ | [Eq.S7] |
| ${CLU}_{k} \sim N_{4}\left( 0, \begin{matrix} {\sigma^{2}}_{C{LU}_{m0}^{urban}} & \rho\left( {CLU}_{m0j}^{urban},{CLU}_{v0j,exp}^{urban} \right)\sigma_{{CLU}_{m0}}^{urban}\sigma_{{CLU}_{v0,exp}}^{urban} & 0 & 0 \\ \ldots& {\sigma^{2}}_{{CLU}_{v0,exp}^{urban}} & 0 & 0 \\ \ldots& \ldots& {\sigma^{2}}_{{CLU}_{m0}^{forest}} & \rho\left( {CLU}_{m0j}^{forest},{CLU}_{v0j,exp}^{forest} \right)\sigma_{{CLU}_{m0}}^{forest}\sigma_{{CLU}_{v0,exp}}^{forest} \\ \ldots& \ldots& \ldots& {\sigma^{2}}_{{CLU}_{v0,exp}^{forest}} \end{matrix} \right)$ | [Eq.S8] |

**References:**

Auld, J.R. & Charmantier, A. (2011). Life history of breeding partners alters age‐related changes of reproductive traits in a natural population of blue tits. *Oikos*, 120, 1129–1138.

Biard, C., Brischoux, F., Meillère, A., Michaud, B., Nivière, M., Ruault, S., *et al.* (2017). Growing in cities: An urban penalty for wild birds? A study of phenotypic differences between urban and rural great tit chicks (*Parus major*). *Front. Ecol. Evol.*, 5, 1–14.

Caizergues, A.E., Grégoire, A. & Charmantier, A. (2018). Urban versus forest ecotypes are not explained by divergent reproductive selection. *Proc. R. Soc. B Biol. Sci.*, 285, 20180261.

Corsini, M., Schöll, E.M., Di Lecce, I., Chatelain, M., Dubiec, A. & Szulkin, M. (2021). Growing in the city: Urban evolutionary ecology of avian growth rates. *Evol. Appl.*, 14, 69–84.

Culina, A., Adriaensen, F., Bailey, L.D., Burgess, M.D., Charmantier, A., Cole, E.F., *et al.* (2020a). Connecting the data landscape of long-term ecological studies: The SPI-Birds data hub. *J. Anim. Ecol.*, 90, 1–14.

Culina, A., Bailey, L.D., Nater, C.R., Vriend, S.J.G. & Visser, M.E. (2020b). Standard protocol for the collection of individual level data.

Dhondt, A.A. (1989). The effect of old age on the reproduction of Great Tits Parus major and Blue Tits P. caeruleus. *Ibis*, 131, 268–280.

Dingemanse, N.J., Both, C., Drent, P.J., Van Oers, K. & Van Noordwijk, a J. (2002). Repeatability and heritability of exploratory behaviour in great tits from the wild. *Anim. Behav.*, 64, 929–938.

European Environment Agency. (2020). Imperviousness Density 2018. https://land.copernicus.eu/user-corner/technical-library/imperviousness-2018-user-manual.pdf

European Environment Agency. (2021). CORINE Land Cover.

Garant, D., Kruuk, L.E.B., Wilkin, T.A., McCleery, R.H. & Sheldon, B.C. (2005). Evolution driven by differential dispersal within a wild bird population. *Nature*, 433, 60–65.

Jarrett, C., Powell, L.L., McDevitt, H., Helm, B. & Welch, A.J. (2020). Bitter fruits of hard labour: diet metabarcoding and telemetry reveal that urban songbirds travel further for lower-quality food. *Oecologia*, 193, 377–388.

Kaiser, A., Merckx, T. & Van Dyck, H. (2016). The Urban Heat Island and its spatial scale dependent impact on survival and development in butterflies of different thermal sensitivity. *Ecol. Evol.*, 6, 4129–4140.

van Overveld, T., Adriaensen, F. & Matthysen, E. (2011). Postfledging family space use in great tits in relation to environmental and parental characteristics. *Behav. Ecol.*, 22, 899–907.

Seress, G., Sándor, K., Bókony, V., Bukor, B., Hubai, K. & Liker, A. (2025). Radio‐tracking urban breeding birds: The importance of native vegetation. *Ecol. Appl.*, 35, e3095.

Seress, G., Sándor, K., Evans, K.L. & Liker, A. (2020). Food availability limits avian reproduction in the city: An experimental study on great tits *Parus major*. *J. Anim. Ecol.*, 89, 1570–1580.

Strubbe, D., Hudin, N.S., Teyssier, A., Vantieghem, P., Aerts, J. & Lens, L. (2020). Phenotypic signatures of urbanization are scale-dependent: a multi-trait study on a classic urban exploiter. *Landsc. Urban Plan.*, 197, 103767.

Svensson, L. (1992). *Identification guide to European passerines*.

Szulkin, M. & Sheldon, B.C. (2008). Dispersal as a means of inbreeding avoidance in a wild bird population. *Proc. R. Soc. B Biol. Sci.*, 275, 703–711.

Tremblay, I., Thomas, D., Blondel, J., Perret, P. & Lambrechts, M.M. (2005). The effect of habitat quality on foraging patterns, provisioning rate and nestling growth in Corsican Blue Tits *Parus caeruleus*. *Ibis*, 147, 17–24.

Uchida, K., Blakey, R.V., Burger, J.R., Cooper, D.S., Niesner, C.A. & Blumstein, D.T. (2021). Urban biodiversity and the importance of scale. *Trends Ecol. Evol.*, 36, 123–131.

Waterschoot, B.O.G., Bataille, G. & Van Dyck, H. (2023). Spatial scale-dependent effects of urbanisation on phenotypic traits in a thermophilous grasshopper. *Behav. Ecol. Sociobiol.*, 77, 1–20.

Wilkin, T.A., Garant, D., Gosler, A.G. & Sheldon, B.C. (2006). Density effects on life-history traits in a wild population of the great tit *Parus major*: analyses of long-term data with GIS techniques. *J. Anim. Ecol.*, 75, 604–615.
